# Protein *N-*glycosylation and *N-*glycan trimming are required for postembryonic development of the pest beetle *Tribolium castaneum*

**DOI:** 10.1038/srep35151

**Published:** 2016-10-12

**Authors:** Tomasz Walski, Els J. M. Van Damme, Nicolas Smargiasso, Olivier Christiaens, Edwin De Pauw, Guy Smagghe

**Affiliations:** 1 Laboratory of Agrozoology, Department of Crop Protection, Faculty of Bioscience Engineering, Ghent University, Ghent, Belgium; 2 Laboratory of Biochemistry and Glycobiology, Department of Molecular Biotechnology, Faculty of Bioscience Engineering, Ghent University, Ghent, Belgium; 3 Mass Spectrometry Laboratory, GIGA-Research, University of Liege, Liege, Belgium

## Abstract

In holometabolous insects the transition from larva to adult requires a complete body reorganization and relies on *N-*glycosylated proteins. *N-*glycosylation is an important posttranslational modification that influences protein activity but its impact on the metamorphosis has not been studied yet. Here we used the red flour beetle, *Tribolium castaneum*, to perform a first comprehensive study on the involvement of the protein *N-*glycosylation pathway in metamorphosis. The transcript levels for genes encoding *N-*glycan processing enzymes increased during later developmental stages and, in turn, transition from larva to adult coincided with an enrichment of more extensively modified paucimannose glycans, including fucosylated ones. Blockage of *N-*glycan attachment resulted in larval mortality, while RNAi of α-glucosidases involved in early *N-*glycan trimming and quality control disrupted the larva to pupa transition. Additionally, simultaneous knockdown of multiple genes responsible for *N-*glycan processing towards paucimannose structures revealed their novel roles in pupal appendage formation and adult eclosion. Our findings revealed that, next to hormonal control, insect post-embryonic development and metamorphosis depend on protein *N-*glycan attachment and efficient *N-*glycan processing. Consequently, disruption of these processes could be an effective new approach for insect control.

*N-*glycosylation or the attachment of an oligosaccharide to asparagine (N) is one of the most important post-translational modifications of proteins (PTM). This process begins in the endoplasmic reticulum (ER) with the transfer of a glycan precursor from a lipid carrier to a N-X-T/S/C consensus sequence (X is any amino acid except proline) in a newly translated protein^1^. Following the oligosaccharide attachment, the nascent *N-*glycoprotein is transported through the ER, to the Golgi apparatus and the *N-*glycan is processed by multiple glycosyl hydrolases and transferases (Fig. 1). The early stages of *N-*glycan processing involve the sequential action of α-glucosidases that remove the three glucose residues capping the immature oligosaccharide to produce the Man_9_GlcNAc_2_ *N-*glycan (See Fig. 1 and captions for glycan nomenclature and abbreviations). These processing steps are conserved among eukaryotes and are crucial for the control of protein folding, quality and ER-associated degradation^2^. *N-*linked glycans can be further processed by class I and class II α-mannosidases, *N-*acetylglucosaminyltransferases, *N-*acetylglucosaminidases and fucosyltransferases to form an array of over 20 different high mannose (containing five and more mannose units) and paucimannose *N-*glycans (containing up to four mannose units). In vertebrates a significant share of the glycans can be extended with GlcNAc, additional galactose, GalNAc and sialic acid residues to form a wide variety of complex *N-*glycans^3^. It has been a long lasting consensus that the ability of insects to produce complex glycans is rather limited. However, recent advances indicated that insects produce a plethora of complex glycans that carry antennae modified with sialic acid, glucuronic acid, T-antigen (Galβ1,3GalNAc), fucosylated GalNAcβ1,4GlcNAc or oligosaccharides substituted with sulfate groups^4^,^5^,^6^,^7^. In contrast to vertebrates, insects usually produce complex *N-*glycans at lower levels compared to high mannose and paucimannose *N-*glycans^8^.

*N-*glycosylation is crucial for protein folding, transport and secretion but may also affect protein activity^2^,^9^. Studies in *Drosophila melanogaster* have shown that *N-*glycosylated proteins are involved in receptor functions, transport or cell adhesion and play important roles in immunity and the central nervous system^10^,^11^,^12^. Notably, multiple proteins implicated in insect metamorphosis have recently been shown to be *N-*glycosylated^13^,^14^,^15^,^16^. However, no research has addressed the intriguing question of a potential role for protein *N-*glycosylation in the development from juvenile to adult life forms.

The life cycle of holometabolous insects such as beetles includes metamorphosis or the transition between morphologically distinct larval, pupal and mature adult stages (Fig. 2.). The induction of this process is controlled by levels of juvenile and ecdysteroid hormones but the understanding of the exact mechanism behind the insect metamorphosis is far from complete^17^,^18^. Especially little is known with regard to involvement of protein post-translational modifications such as *N-*glycosylation in the execution of the metamorphosis. *Tribolium castaneum*, the red flour beetle, appears to be an outstanding model to study these aspects owing to a completely sequenced genome and systemically working RNA interference (RNAi). Through injection of a mixture of dsRNAs it is possible to silence up to three genes simultaneously, which allows to uncover phenotypes masked by functionally redundant genes in single knock-downs. Additionally, differences in *Tribolium* development including the formation of wing covers (elytra), which are not present in fruit fly, enable the investigation of novel gene functions^19^. Importantly, *T. castaneum* is a pest, thus deeper insight into the biochemistry of its metamorphosis can bring relevant ideas for novel insect control strategies replacing currently used synthetic ones that may pose a risk to human health or the environment^20^,^21^.

In this study we address the question of the involvement of protein *N-*glycosylation and *N-*glycan trimming in post-embryonic development and metamorphosis in *T. castaneum*. For that purpose we studied the expression patterns of genes putatively involved in the major steps of the protein *N-*glycosylation pathway that is transfer of the precursor oligosaccharide to a polypeptide, early *N*-glycan processing as well as maturation of the oligosaccharide towards paucimannose and complex structures. Secondly, we analyzed the metamorphosis-related changes in the *N-*glycan composition. Finally, we used RNAi to explore roles of each part of the *N-*glycosylation pathway in the post-embryonic development and metamorphosis of *T. castaneum* beetles. Here we show, for the first time, that the transition from larva to adult was associated with the enrichment of extensively modified *N-*glycan forms. The observed changes in *N-*glycome composition were driven by the developmentally regulated expression of genes coding for *N-*glycan processing enzymes. Furthermore, the arrest of the *N-*glycan attachment blocked larval growth, while disruption of *N-*glycan processing revealed novel *N-*glycosylation roles in pupa and adult development, particularly in elytra formation. Based on our data, we postulate that efficient *N-*linked glycan processing is a key for successful post-embryonic development and metamorphosis in the red flour beetle.

## Results and Discussion

### Expression of genes putatively involved in *N-*glycosylation changes during post-embryonic development

**Figure 1:**
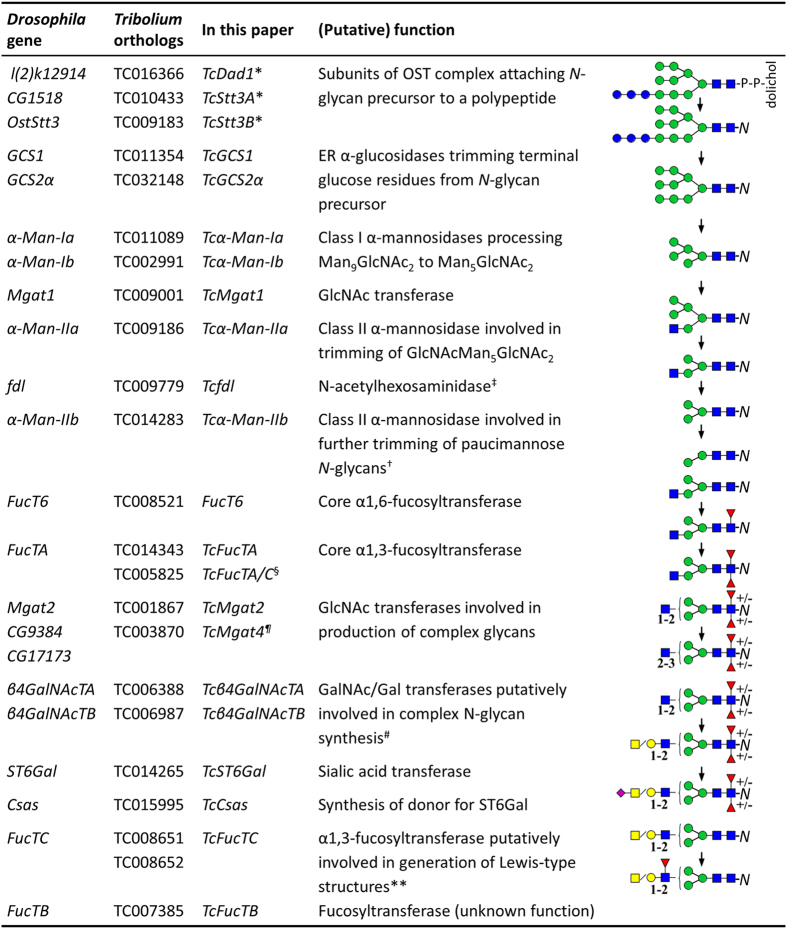
Overview of *T. castaneum* genes putatively involved in *N-*glycosylation, studied in this work. 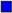
 or GlcNAc – *N-*acetylglucosamine, 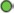 or Man – mannose, 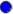 or Glc – glucose, 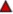 or Fuc – fucose, 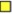 or GalNAc – *N-*acetylgalactosamine, 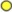 or Gal – galactose, 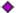 sialic acid, ***N*** – asparagine in a polypeptide chain, P-P-dolichol – lipid carrier of *N-*glycan precursor. *Named based on homology with human OST subunits, ^‡^Enzyme encoded by *fdl* can also remove terminal *N-*acetylhexosamine residues from biantennary LacdiNAc or core fucosylated glycans^23^, ^†^*Drosophila* α-Man-IIb may function downstream to α-Man-IIa to produce paucimannose glycans^25^, but can also trim single α1,2 mannose residues from Man_9-8_GlcNAc_2_ and process Man_5_GlcNAc_2_ into Man_3_GlcNAc_2_^22^, ^§^TC005825 has similar evolutionary relationship with *Drosophila* FucTA and FucTC (see Supplementary Fig. S1i), ^¶^Named based on human ortholog MGAT4, ^#^homolog from moth *Trichoplusia ni* next to GalNAc may transfer Gal as well^24^. Insect *β*4GalNAcT’s may also be involved in synthesis of glycolipids, **The function of putative FucTC was based on the activity of the ortholog from mosquito (*Anopheles gambiae*)^6^.

**Figure 3:**
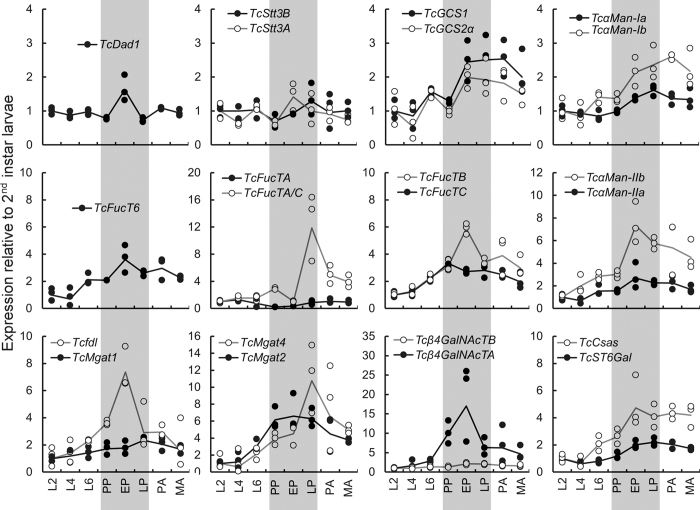
Expression of genes involved in *N-*glycosylation pathway changes during post-embryonic development. L2, L4, L6 – 2^nd^, 4^th^, 6^th^ instar larvae; PP, EP, LP – pre-, early, late pupa; PA, MA – pharate, mature adults. Relative gene expression was quantified with qRT-PCR using *TcRpL32* and *TcRpS6* as reference genes and normalized to L2 expression level. All data points are shown (n = 3). Lines connect the average values. Note the highest expression of most genes during pupal periods (marked in grey). See Supplementary Table S1 for statistics.

To identify putative genes involved in *N-*glycosylation in *Tribolium* we used blast tools with *D. melanogaster* sequences as queries and constructed phylogenetic trees to confirm the true homology of the identified sequences. These analyses revealed that the genome of *T. castaneum* encodes a similar array of genes (Fig. 1, Supplementary Figs S1 and S2) putatively involved in the *N-*glycan attachment and processing as previously described for the model insect *D. melanogaster*^11^,^22^,^23^,^24^,^25^, except for the class I α-mannosidase c, which appears to be absent in *Tribolium*. To gain a first insight into the importance of *N-*glycosylation for *Tribolium* post-embryonic development, we analyzed the expression patterns of 22 of the identified genes. For that we used qRT-PCR to estimate changes in the transcript abundances at eight life stages between 2^nd^ instar larvae and mature adults (Fig. 3 and Supplementary Table S1). We observed statistically significant differences in mRNA expression between life stages for all of the tested genes (ANOVA, P < 0.05), except for *TcStt3B* (P = 0.547). Interestingly, nineteen of the genes showed the highest expression levels during the pupal stage (twelve in early and seven in the late pupae). In contrast, there was only one gene *TcFucTA*, encoding a putative α-1,3-fucosyltransferase, which showed the highest expression during the larval stage (4^th^ instar). mRNA levels of most of the genes involved in *N-*glycan processing in the ER and Golgi were higher in mature adults compared to 4^th^ instar larvae (midpoint of larval development, Supplementary Table S1). Notably, putative class I and II α-mannosidases, key players in the trimming of *N-*glycans, were 1.5 to 2.5 fold upregulated, while the expression of genes potentially involved in the synthesis of complex *N-*glycans (putative *TcMgat2*, *TcMgat4*, *Tcβ4GalNAcTA*, *Tcβ4GalNAcTB* and *TcST6Gal*) was 2–6 fold higher. The link between metamorphosis and glycan processing genes was also found in large-scale transcriptome analyses in *D. melanogaster* that showed increased expression of these genes in pupae and adults compared to larvae^26^,^27^. Although the levels of upregulation for *N-*glycan processing genes in adults were moderate they were consistent across the *N-*glycan processing pathway. In contrast, genes putatively involved in the attachment of the *N-*glycan precursor to a polypeptide (*TcDad1*, *TcStt3A*, *TcStt3B*) were expressed at similar levels in larvae and adults. Thus we hypothesized that the transition from larvae to adults (metamorphosis) is associated with a shift in the *N-*glycan composition towards more processed forms.

### Adults contain more processed *N-*glycans than larvae

**Figure 4:**
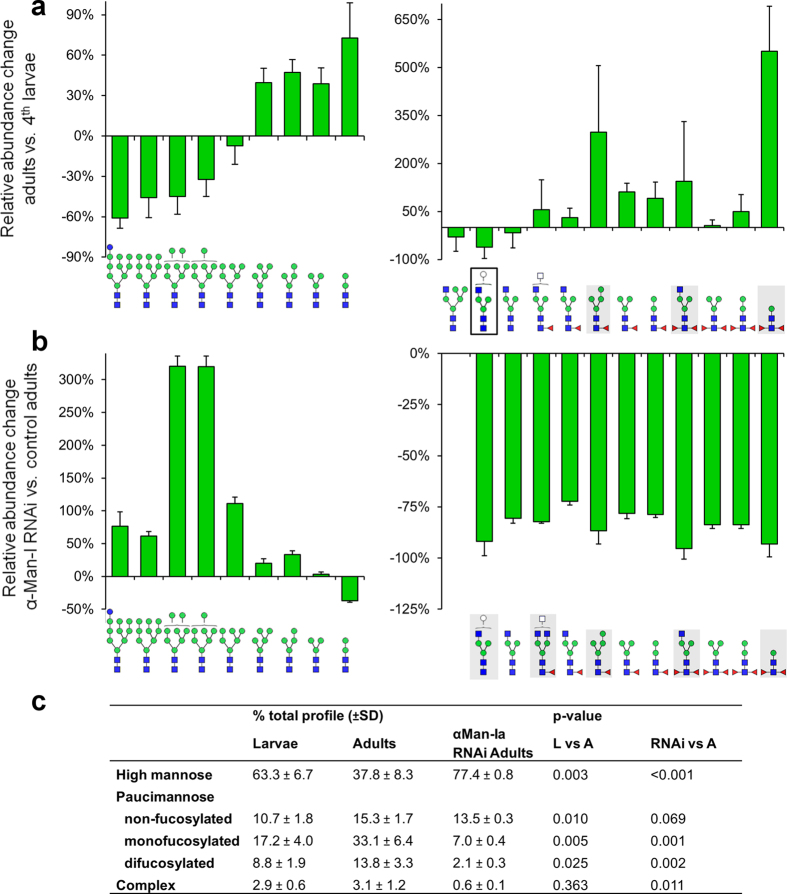
Metamorphosis is associated with enhanced *N-*glycan processing, which can be disrupted by α-Man-Ia RNAi. Proteins were extracted from whole 4^th^ instar larvae or mature adults. *N-*glycans were released using PNGase A, labeled with 2-AB and analyzed using MALDI-MS. (**a**) Relative changes in adult *N-*glycan abundance compared to average *N-*glycan abundance in 4^th^ instar larvae. (**b**) Relative changes in *N-*glycan abundance in adults eclosed after silencing of *Tc*α*-Ma*n-I*a* compared to wild type adults. Left: mannose series *N-*glycans, right: insect complex, hybrid and fucosylated *N-*glycans. Error bars are SD (n = 4). Glycans in black outline were below 0.1% of total pool in at least two adult samples, grey boxes indicate those below 0.1% in larvae (**a**) or RNAi adults in (**b**), GlcNAcMan_5_GlcNAc_2_ was not detected in RNAi samples. White circle and square indicate undetermined hexose (mannose or galactose) and N-acetylhexosamine (GlcNAc or GalNAc) residues, respectively. (**c**) Abundances and statistical differences of different glycan groups, p-values were obtained using independent samples t-test. See Fig. 1 for *N-*glycan symbol description and Supplementary Table S2 for detailed *N-*glycan quantitation.

To verify if metamorphosis is associated with enhanced *N-*glycan processing we compared the composition of the *N-*glycans attached to proteins from mature *Tribolium* adults and 4^th^ instar larvae. *N-*glycans were cleaved from the peptide backbone using either PNGase F (inactive on *N-*glycans containing core α1,3-fucose) or PNGase A (cleaving all *N-*glycans regardless of fucosylation), and then subjected to permethylation or 2-aminobenzamide (2-AB) labeling followed by MALDI-TOF analysis. Each of the approaches yielded a similar profile of identified *N-*glycans, except for peaks corresponding to difucosylated glycans which were observed only in samples treated with PNGase A. Therefore, the *N-*glycans released with PNGase A and labeled with 2-AB were used for relative quantifications. For the statistical analyses only the structures repeatedly detected at levels above 0.1% of total *N-*glycan pool in at least one of the tested stages were selected. Both larval and adult *N-*glycomes yielded major glycan structures as previously reported for other insects^4^,^28^,^29^ consisting mainly of high- and paucimannose *N-*glycans with only a minor fraction of complex glycans (containing at least one GlcNAc residue extending the Man3GlcNAc structure; Fig. 4a,c, Supplementary Table S2, Supplementary Fig. S3). However, in line with the elevated expression of the genes encoding *N-*glycan processing enzymes, the composition of the adult *N-*glycome was shifted towards more trimmed *N-*glycans. Larval samples were dominated by Man_9_GlcNAc_2_ *N-*glycan (40.7 ± 7.4% of total profile) and the second most abundant *N-*glycan was Man_2_GlcNAc_2_Fuc (11.5 ± 3.1%), while in adults both of these glycans were detected at similar levels (22.0 ± 6.0% and 21.9 ± 5.8% of all *N-*glycans, respectively). In larvae each of the high mannose structures (Man_5–9_GlcNAc_2_) was up to 2 times more abundant compared to adults. In total almost 2/3 of the larval glycans were of high mannose type while these glycans constituted less than 40% of the adult sample (P = 0.003). Consequently, all types of paucimannose glycans were upregulated in adults compared to larvae. Among them, oligosaccharides carrying a single fucose residue accounted for 33.1 ± 6.4% of the total *N-*glycan pool in mature adults compared to 17.2 ± 4.0% in larvae (P = 0.005). Also non-fucosylated paucimannose glycans were approximately 50% more abundant in adults (P = 0.010). Interestingly both life stages contained high levels of difucosylated *N-*glycans. Taken together difucosylated *N-*glycans were significantly more abundant in adults (8.8 ± 1.9% vs. 13.8 ± 3.3%, P = 0.025). A particular glycan in this group was extensively trimmed ManGlcNAc_2_Fuc_2_, which was consistently detected in adult at levels near 1% of the total *N-*glycan content but in larvae it was found in only one out four biological replications. Changes in the total *N-*glycan profile between post-embryonic life stages have not been reported so far for other insects but were shown for the nematode *C. elegans*^30^ suggesting that glycosylation dynamics during development may be a widespread phenomenon.

The composition of the *N-*glycomes for both *T. castaneum* larvae and adults, differs from that of fruit fly embryos and adults or mosquito larvae, in which lower amounts of Man_9_GlcNAc_2_ and Man_2_GlcNAc_2_Fuc but higher levels of Man_3_GlcNAc_2_Fuc and Man_5_GlcNAc_2_ were found^4^,^31^,^32^. Yet, the most striking difference between *Drosophila* and *Tribolium* was the content of difucosylated glycans. In the fruit fly difucosylated glycans represented below 1% of the total *N-*glycan profile and appear to be enriched in neural tissue^33^. In contrast markedly higher levels of these glycans appear in beetles, representing up to 17%. It is an intriguing issue whether these high-abundant difucosylated glycans in *Tribolium* are also predominantly produced in neural tissues or, possibly, are synthesized more ubiquitously in other tissues as well. Since the genetic make-up of the glycosylation pathway is similar between coleopteran and dipteran insects, the observed differences in the *N-*glycan composition may result from divergent expression, activity or substrate specificity of homologous enzymes.

Although the expression of most enzymes potentially involved in complex glycan synthesis was significantly enhanced in adults compared to 4^th^ instar larvae, there were no differences between the complex *N-*glycan content in larvae and adults (3.1 ± 1.2% and 2.9 ± 0.6% of the total profile, respectively; P = 0.273). The most elaborated structure in the *Tribolium* glycome was GlcNAc_2_Man_3_GlcNAc_2_Fuc and no sialylated glycans were detected in any of the life stages. This might be due to the activity of *fdl (β*-*N-*acetylglucosaminidase) which redirects the pathway towards synthesis of paucimannose glycans and reduces the content of complex glycans^28^. Furthermore, the highest expression levels of *TcCsas* and *TcST6Gal*, putatively responsible for sialic acid activation and attachment, were still 100- and 20-fold lower compared to genes encoding earlier steps of the pathway which is in agreement with the low and spatially restricted expression of the *D. melanogaster* orthologs^34^.

### RNAi of genes involved in the early steps of the *N-*glycosylation pathway blocks larval development

Elevated expression of genes encoding enzymes responsible for *N-*glycan trimming and modification during later developmental stages as well as enrichment of more processed *N-*glycans in adults suggested that *N-*glycan processing is implicated in metamorphosis. To verify this hypothesis we knocked-down an array of genes involved in *N-*glycosylation and studied the consequences for insect development. Injection of specific dsRNAs into *Tribolium* larvae induces RNAi-mediated gene silencing that persists throughout metamorphosis and in the mature adults. In our hands injection of gene-specific dsRNA into 4^th^ instar larvae generally resulted in an 80–95% reduction of transcript abundance (Supplementary Fig. S4).

**Figure 5:**
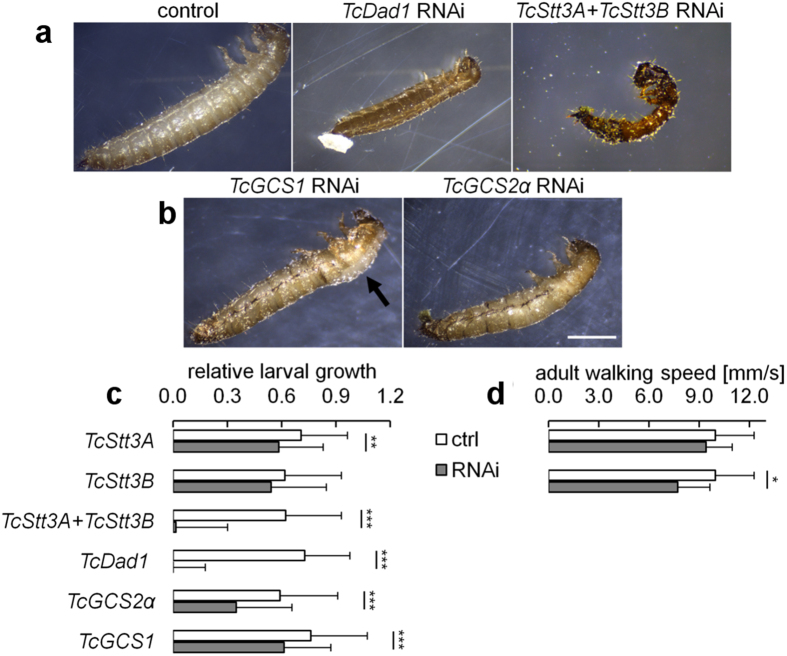
Silencing of genes involved in *N-*glycan attachment and early trimming disrupts larval development and prevents pupation. 4^th^ instar larvae were injected with appropriate dsRNAs to induce gene silencing or with dsGFP in case of control. (**a**) RNAi of *TcDad1* and double RNAi of *TcStt3A* and *TcStt3B* isoforms resulted in early larval mortality. (**b**) RNAi of *TcGCS1* and *TcGCS2*α resulted in mortality during last larval and prepupal stages. Black arrow indicates dorsal split in the cuticle suggesting that pupation was initiated before the larvae died. (**c**) RNAi effects on larval growth (n = 51, 61, 43, 56, 58, 61 – top to down). (**d**) RNAi of *TcStt3B* but not of *TcStt3A* induced minor reduction of adult walking speed, n = 13. Error bars are SD, ***P < 0.001, **P < 0.01, *P < 0.05.

Silencing of *TcDad1*, encoding an essential subunit of the OST complex catalyzing the attachment of the *N-*glycan precursor to a polypeptide, completely inhibited larval growth and caused 96.4% mortality during the larval stage (Table 1, Fig. 5a). The OST contains either the Stt3A or Stt3B isoform of the catalytic subunit determining activity of the complex^35^,^36^. When we silenced either of these subunit isoforms we observed only weak phenotypes: minor reduction of larval growth or adult movement speed. This is in line with the lack of severe phenotypes caused by mutations and knockdowns of Stt3A or Stt3B subunits in *Arabidopsis* but is in contrast to lethality caused by mutation in a *OstStt3,* ortholog of *Stt3B,* in *Drosophila*^37^ and single stt3 gene in *C. elegans*. It has been shown that mammalian Stt3A and B isoforms have partially redundant activities^38^, so we asked if this can also be the case in *T. castaneum*. Co-silencing of both *Tribolium Stt3* isoforms resulted in high larval lethality similar to that caused by RNAi of *Tribolium Dad1* (Fig. 4a,c), coding for an essential subunit of OST^39^. This supports functional compensation between *TcStt3A* and *TcStt3B* and indicates that *N-*glycan attachment is crucial for larval growth and survival.

The first step of *N-*glycan maturation of glycoproteins involves the action of two α-glucosidases, GCS1 and GCS2, which act in the ER to sequentially remove three glucose residues from the immature *N-*linked glycan to form the Man_9_GlcNAc_2_ structure^1^. Silencing of *TcGCS1* and *TcGCS2*α resulted in a reduced larval growth by 19% (P = 0.006) and 41% (P < 0.001), respectively, and prevented pupation (Fig. 4b–d). During the normal course of development fully grown larvae develop into a characteristic crescent-shape immobile form or prepupae. After several days the prepupal cuticle breaks open to allow the pupa to emerge. Approximately 90% of *TcGCS1* and *TcGCS2*αRNAi beetles died during the prepupal stage (Table 1). In most of these dead prepupae we observed a dorsal split in the old cuticle, which was not followed by emergence of the pupa. This suggested that the pupation was initiated but the beetles died before the process could be completed. A comparable phenotype was observed for RNAi of TC012278^40^, a putative *Tribolium* ortholog for calreticulin, one of the key players in *N-*glycoprotein folding^2^. *N-* Interestingly, knockdown of the GCS1 ortholog in *C. elegans* affected worm growth, movement and viability, which was attributed to alteration of the entire *N-*glycome, compromised protein quality control and chronic ER stress^41^. Based on the evidence from other organisms, the GCS1 and GCS2α orthologs in *Tribolium* are, most likely, involved in *N-*glycoprotein quality control which is important throughout the life cycle of the beetle. However, RNAi of these genes resulted in minor larval growth defects but blocked the transition from prepupa to pupa. This together with an observed increase in the transcript levels of both genes between the prepupal and early pupal stage (Fig. 3) suggests the particular requirement for efficient *N-*glycoprotein processing and quality control in the early phase of the metamorphosis. The only studies of α-glucosidases in *Drosophila* involved transgenic RNAi restricted to notum (dorsal part of thoracic segment). This did not prevent pupation but resulted in some lethality before the end of the pupal stage^42^ which may suggest timing differences between the fly and the beetle in the requirement for first steps of *N-*glycan maturation.

### Knockdown of genes involved in trimming of high-mannose *N-*glycans affects pupal and adult development

**Figure 6:**
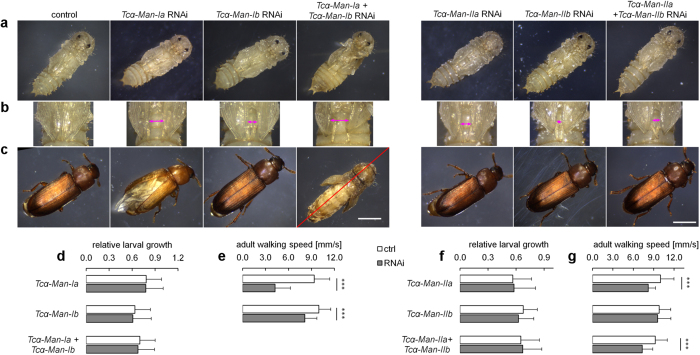
Silencing of α-mannosidases affects pupa and adult development. 4^th^ instar larvae were injected with appropriate dsRNAs to induce gene silencing or with dsGFP in case of control. (**a**) Single knockdowns by RNAi of class I and II α-mannosidases induced minor changes in position and shape of pupal elytra, while double knockdown of *Tc*α*-Man-Ia* and *Tc*α*-Man-Ib* caused severe pupal malformations. (**b**) Zoomed view on pupal elytra. (**c**) Silencing of *Tc*α*-Man-Ia* disrupted adult elytra development, but double RNAi of *Tc*α*-Man-Ia* and *Tc*α*-Man-Ib* completely prevented adult eclosion. Double RNAi of *Tc*α*-Man-IIa* and *Tc*α*-Man-IIb* did not increase severity of phenotypes compared to single knockdowns. (**d**) and RNAi of class I α-mannosidases did not affect larval growth (n = 61, 53, 53). (**e**) RNAi of *Tc*α*-Man-Ia* and of *Tc*α*-Man-Ib* decreased adult mobility (n = 15, 22). No living adults eclosed after double RNAi of *Tc*α*-Man-Ia* and *Tc*α*-Man-Ib.* (**f**) RNAi of class II α-mannosidases did not affect larval growth (n = 60, 52, 61). (**g**) RNAi of *Tc*α*-Man-IIa* and double RNAi of *Tc*α*-Man-IIa* and *Tc*α*-Man-IIb* decreased adult mobility (n = 24, 29, 29). Scale bar is 1 mm in (**a**) and (**c**) and 0.5 mm in (**b**). Error bars are SD, ***P < 0.001 (t-test).

Following the action of the α-glucosidases the Man_9_GlcNAc_2_ can be trimmed to Man_5_GlcNAc_2_ by class I α-mannosidases. The *Tribolium* genome encodes two enzymes potentially involved in these reactions: α-Man-Ib thought to specifically cleave a single mannose residue from the middle antenna of a glycan and α-Man-Ia cleaving additional three mannoses^22^. In contrast to silencing of the genes implicated in the initial steps of the *N-*glycosylation pathway, knock-down of *Tc*α*-Man-Ia* and *Tc*α*-Man-Ib* did not affect survival, larval growth or time of pupation but rather affected the morphology of the pupae (Fig. 6a). In control pupae, the elytra (wing covers) almost completely cover the abdomen and the third pair of legs. In contrast, in insects treated with dsRNA for class I α-mannosidases the elytra were miss positioned (Fig. 6b, Supplementary Fig. S6). The gap between the elytra was 0.31 ± 0.04 and 0.15 ± 0.03 mm in the case of *Tc*α*-Man-Ia* and *Tc*α*-Man-Ib* RNAi, respectively, compared to 0.06 ± 0.03 mm in the control beetles (both P < 0.001). Silencing of α-Man-Ia gene also yielded shorter pupal elytra: 1.31 ± 0.07 mm compared to 1.42 ± 0.06 mm in the control group (P < 0.001) and induced effects on the adult appendages. The adult elytra were wrinkled and not closed, revealing underlying flight wings which appeared smaller and crumbled (Fig. 6c, Supplementary Fig. S7). In both cases RNAi reduced adult walking speed by over 50% for *Tc*α*-Man-Ia* (P < 0.001) and approx. 20% for *Tc*α*-Man-Ib* (P = 0.003).

In *D. melanogaster*, mutations of the α*-Man-*n-I*a* ortholog are also not lethal, but affect the immunity, nervous system, wings and eyes^12^,^25^,^43^. These relatively weak phenotypes were explained by the presence of an alternative N-glycan processing pathway inferred from the observation that N-glycan processing is not blocked completely in α-Man-Ia mutant flies^44^. It was suggested that an unusual class II α-mannosidase (α-Man-IIb or α-Man-III) could be involved in this alternative trimming route^22^,^45^.

Our analysis of adult N-glycans isolated after knockdown of *Tc*α*-Man-Ia* revealed a clear accumulation of high mannose N-glycans and a reduction but not total absence of paucimannose *N-*glycans (Fig. 4b,c, Supplementary Table S2, Supplementary Fig. S3). Man_9_GlcNAc_2_ was still the most prevalent glycan and its relative abundance was increased by 60% compared to control adults (P = 0.010). In total high mannose *N-*glycans amounted to 77.4 ± 0.8% of all *N-*glycans in *Tc*α*-Man-Ia* RNAi beetles compared to 37.8 ± 8.3% in the control (P < 0.001). This lack of complete blockage of *N-*glycan processing is in line with the hypothetical partially compensatory activity of α-Man-IIb.

Surprisingly, the amount of Man_5_GlcNAc_2_, the end product of α-Man-Ia, was not reduced but enriched by ~20% (P = 0.013). However, the glycan that is formed by the addition of a terminal GlcNAc, GlcNAcMan_5_GlcNAc_2_, was below the detection limit in *Tc*α*-Man-Ia* knockdown adults. Consequently, the prevalence of each of the complex and core fucosylated oligosaccharides was reduced by 3 to 20 fold. In total, monofucosylated, difucosylated and complex *N-*glycans were decreased by 4.7, 6.6 and 5.2 fold, respectively (P = 0.001, 0.002 and 0.011, respectively). In contrast, the content of non-fucosylated paucimannose *N-*glycans in *Tc*α*-Man-Ia* RNAi adults was not significantly different from that in the control adults (P = 0.069, Fig. 4c). However, levels of particular *N-*glycans among this group were differentially affected. For instance, the Man_4_GlcNAc_2_ was enriched by ~35% (P = 0.001), the Man_3_GlcNAc_2_ was unchanged (P = 0.294) and the Man_2_GlcNAc_2_ was downregulated by ~40% (P = 0.007) in *Tc*α*-Man-Ia* RNAi adults.

Reduced content of complex and paucimannose *N-*glycans could have been caused by more pronounced activity of α-Man-IIb in α*-Man-*n-I*a* RNAi beetles. Similar mechanisms have been observed in CHO cells. In this cell line overexpression of α-Man-IIx, a mammalian ortholog of α-Man-IIb, lead to a reduction of the complex *N-*glycan content caused by an accumulation of Man_3_GlcNAc_2_ and Man_4_GlcNAc_2_^46^. These two glycans are not efficiently processed by the subsequent enzyme Mgat1^47^, which catalyzes a key step for fucosylation and complex glycan synthesis in invertebrates^48^,^49^,^50^.

Remarkably, double knockdown of *Tc*α*-Man-Ia* and *Tc*α*-Man-Ib* resulted in more pronounced pupa malformations and in an arrest of wing and leg development implying some functional overlap between the genes (Fig. 5, Supplementary Fig. S7). All double RNAi insects that reached the pupal stage (~73%) died before the adult eclosion was completed. It is thought that Man-Ib is mainly involved in folding of *N-*glycosylated proteins in the ER (or ER-derived quality control vesicles) while Man-Ia is responsible for trimming of high mannose *N-*glycans on proteins in the Golgi^51^,^52^. Yet, the lack of mortality in case of single knockdowns and severe phenotypes after double knockdown of both class I mannosidase genes indicated that the proteins they encode might have, at least partially, overlapping functions. Moreover, the silencing of class I mannosidases produced no discernable phenotypes during the larval stage. Clear morphological alterations could be observed starting from the early pupal stage, which is in line with the increase of mRNA levels for both genes between the larval and pupal stage. These observations along with the glycan analysis data implies that trimming of high mannose *N-*glycans is necessary for metamorphosis and that *Tc*α*-Man-IIb* does not efficiently contribute to this process in the absence of both *Tc*α*-Man-Ia* and *Tc*α*-Man-Ib*.

To verify whether the observed wing and elytra malformations could be due to the effect of RNAi on development of the wing imaginal discs, we repeated the knockdown experiments in the *pu11 nub* enhancer trap line, which allowed to follow proliferation of the wing discs by EYFP expression^53^. However, silencing of the *Tc*α*-Man-Ia* gene alone or in combination with *Tc*α*-Man-Ib* caused no obvious effects on the formation of the wing imaginal discs (Supplementary Fig. S6d), which suggested that class I α-mannosidases are required in later phases of wing development.

### Paucimannose *N-*glycans are involved in elytra development and adult movement

Silencing of Mgat1, α-Man-IIa, α-Man-IIb and fdl genes, involved in the production of paucimannose glycans, did not affect larval growth, mortality, or timing of pupation and adult eclosion. Similarly to RNAi of *Tc*α*-Man-Ia* and *Tc*α*-Man-Ib* we observed effects on elytra and minor effects on pupal elytra length and position (Fig. 6, Supplementary Figs S5 and S6). Additionally, reduced expression of *TcMgat1* and *Tc*α*-Man-IIa* impaired adult movement speed by approx. 30% and 20%, respectively (P < 0.001).

RNAi of α*-Man-IIa* in *D. melanogaster* was lethal^54^ while genes coding for Mgat1, α-Man-IIa, α-Man-IIb and Fdl are not required for fruit fly survival but were implicated in nervous system development, locomotor behavior, longevity and immunity^8^,^12^,^28^. Thus we believe that the reduced mobility of RNAi beetles indicates evolutionary conserved roles of these genes in the nervous system, while the wing cover phenotypes indicate novel roles of *N-*glycosylation in the development of elytra. The sequential action of Mgat1, α-Man-II and fdl may also be bypassed by the activity α-Man-IIb. However, when we co-silenced *Tc*α*-Man-IIa* and *Tc*α*-Man-IIb* we did not observe any enhanced phenotypes compared to single knockdowns, which could confirm the lack of a functional overlap between the enzymes. However, since the transcript abundances in this experiment were only reduced approx. 20% compared to the control levels, the silencing effect was probably not sufficient to block enzyme activity.

So far only approximately 100 *N*-glycosylated proteins have been identified in *T. castaneum*^15^. In *D. melanogaster* the list of known *N-*glycoproteins counts over 1000 entries^10^,^14^,^55^. Among these there are multiple proteins which are involved in the post-embryonic development and metamorphosis. Consequently, some of the observed RNAi phenotypes could be caused by impaired protein function resulting from incomplete processing of *N*-glycans that decorate these proteins. Several of the insect glycoproteins were implicated in development-related signaling, for instance juvenile hormone esterase, insulin receptor A or Smoothened^14^,^15^. Blocked *N-*glycosylation could prevent transport of these proteins to the cell membrane and consequently it could impair their function in the regulation of growth and organ development^56^,^57^. Another group of interesting *N-*glycosylated proteins in *T. castaneum* and *D. melanogaster* includes proteases and chitinases, which may be involved in the recycling of the larval tissues^16^,^58^,^59^ as well as chitin synthases and integrins which are involved in the restructuration of the tissue and the development of new pupal and adult organs, such as wings and wing covers^60^,^61^. Multiple of these proteins fulfill their role after being transported out of the cell^16^,^62^. It has been shown in *Trichoplusia ni* (cabbage looper) cells that the secreted proteins contained predominantly paucimannose or complex *N-*glycans while membrane proteins carried high mannose *N-*glycans^63^. Thus it seems likely, that the processing by Mgat1, α-Man-IIa, Fdl and α-Man-IIb, resulting in paucimannose *N-*glycans, is necessary for the extracellular localization of the glycoproteins involved in tissue remodeling during the metamorphosis. Fasciclin2 and Laminin A were also reported to be *N-*glycosylated both in the red flour beetles and in the fruit flies^15^,^55^. These proteins are involved in axon guidance, nervous system development and locomotor activity^64^,^65^ Thus the observed locomotor phenotypes in adult beetles, resulting from RNAi-mediated disruption of *N-*glycan processing, could be linked to the impairment of the activity of these proteins. Verification of these hypotheses requires a study focused on the localization and activity of glycoproteins after silencing of *N-*glycan processing pathway components.

### RNAi of genes involved in complex *N-*glycan synthesis and fucosylation caused minor effects

Alternatively to the production of paucimannose oligosaccharides, *N-*glycans can be extended with GlcNAc, GalNAc, Gal and negatively charged sugars (glucuronic and sialic acids) to form so-called complex structures. However, in line with minute amounts of complex *N-*glycans found by mass spectrometry, we did not observe any appreciable phenotypes caused by the knockdown of genes putatively responsible for their synthesis, except for a minor (~20%) decrease in larval growth observed after injection of dsRNA for *Tcβ4GalNAcTA* (Supplementary Fig. S8). This is in contrast with neurophysiological defects and abnormal movement caused by mutations of *ST6Gal* and *β4GalNAcTA* in *Drosophila*^34^,^66^.

In an attempt to block the GlcNAc addition and prevent complex glycan synthesis completely, we injected larvae with a mixture of dsRNAs for *TcMgat1*, *TcMgat2* and *TcMgat4.* This resulted in pupal elytra phenotypes and adult movement speed reduction similar to the effects observed when *TcMgat1* was silenced alone (Supplementary Figs S5 and S6).

Finally, we silenced the *Tribolium* orthologs of the *Drosophila* α1,6-fucosyltransferase (*TcFucT6*) and two putative α1,3-fucosyltransferases (*TcFucTA* and *TcFucTA/C*). Knockdown of these genes did not cause any effects. Although in the case of the *FucT6* gene the absence of a phenotype could probably be due to the low knockdown efficiency (37 ± 9%), the RNAi of the genes *TcFucTA* and *TcFucTA/C* was successful (80 ± 3% and 85 ± 2% reduction, respectively). Hence the lack of phenotypes can possibly be explained by the presence of additional fucosyltransferase genes with redundant functions (*FucTB*). We cannot draw firm assumptions about the functional implications of *N-*glycan fucosylation for post-embryonic development; it is evident that the functions and synthesis of paucimannose glycans requires further investigation.

### Protein *N-*glycosylation in fly and beetle: similar yet different

Even though phylogenetic analyses revealed a high degree of conservation between *Drosophila* and *Tribolium* protein *N-*glycosylation pathways, our analyses yielded some notable functional differences and novel observations. Contrary to *Drosophila* in which a mutation of Stt3B ortholog is lethal^37^, both *Tribolium* Stt3A and Stt3B genes appear to be able to compensate for the lack of the other. Unperturbed expression of *GCS1* and *GCS2*α appears to be vital for larval growth and transition into pupa in *Tribolium* while in *Drosophila* these genes appear to be needed for pupa-adult transition^42^. Additionally, the *N-*glycan profile after *Tc*α*-Man-Ia* silencing indicated that even though there is a significant redundancy between *N-*glycan processing enzymes, knockdown of a single gene can disrupt the entire pathway including the fucosylation reactions occurring far downstream. Moreover, simultaneous silencing of both class I α-mannosidases indicated for the first time that Man_9_GlcNAc_2_ on glycoproteins, needs to be further trimmed for the metamorphosis to be completed. Locomotor phenotypes, similar to those observed in *Drosophila*^8^ suggest evolutionary conserved functions of paucimannose *N-*glycans in neuromuscular development, while wing cover phenotypes indicate novel roles for *N-*glycosylation in development of these appendages. Intriguingly, difucosylated glycans, which are rare in *Drosophila*^33^, are among the most abundant ones in beetle, which suggests their much broader distribution and potentially divergent functions.

Finally, silencing of genes that are putatively involved in the *N*-glycan extension with GalNAc and sialic acid had no effect on locomotor behavior of the beetles which is contrary to *Drosophila* mutants^34^,^66^. This suggests that complex glycans are not primarily implicated in the post-embryonic development but rather in the embryonic development or possibly in the response to stress stimuli.

The biochemical basis for the observed differences in the *N-*glycosylation profile is still an open question. A first possibility could be that the glycoproteins expressed during metamorphosis and in adults would have their specific *N-*glycosylation patterns different from those expressed in larval stages. Such an influence of protein structure on the final form of the *N-*glycan has been demonstrated for single insect proteins^67^,^68^. However, if the global change of the *N-*glycan profile would rely mostly on the protein structural features it would not require the observed changes in mRNA levels of *N-*glycan processing genes. A second mechanism emerges from this work in which the increased expression of *N-*glycan processing genes drives an *N-*glycosylation shift of the proteins expressed throughout all life stages and this, in turn, allows them to mediate metamorphosis specific functions. Previous analysis of adult *T. castaneum* glycoproteins revealed that Fasciclins, Laminins, Catalases and V-type ATPases are *N-*glycosylated^15^. Interestingly, RNAi of these proteins in beetle larvae or pupae prevented metamorphosis or caused defects in appendage development, which further supports the latter hypothesis^40^. Probably, both mentioned mechanisms may contribute to shift of the *N-*glycosylation profiles, yet, detailed understanding of this phenomenon would require an exhaustive comparison of *N-*glycoproteins from larvae, pupae and adults.

**Figure 7:**
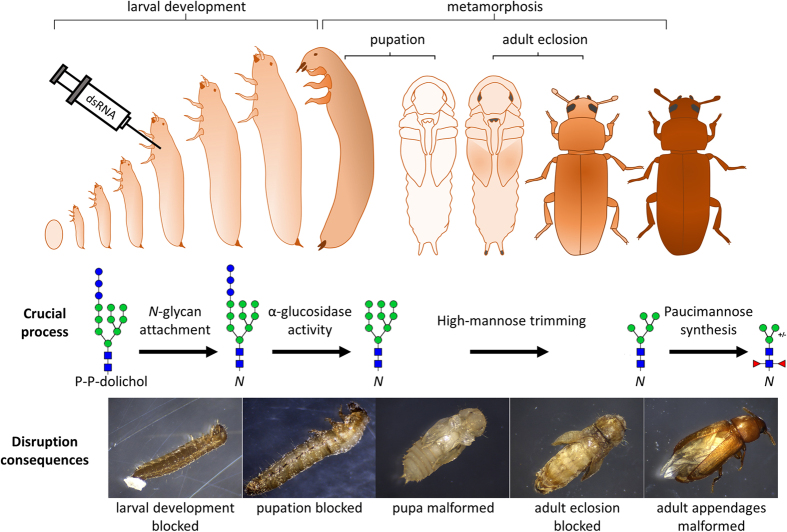
Overview of the involvement of the protein *N*-glycosylation and key steps of *N*-glycan processing in the post-embryonic development of the red flour beetle *T. castaneum* demonstrated by dsRNA mediated gene knockdown (RNAi). Blocked protein N-glycosylation resulted in the arrest of larval development. Knockdown of α-glucosidases involved in early glycan trimming blocked larva to pupa transition (pupation). RNAi of class I α-mannosidases involved in trimming of high-mannose *N*-glycans caused pupa malformation and prevented adult eclosion. Knockdown of genes responsible for production of paucimannose *N*-glycans resulted mainly in malformation of pupal and adult appendages. See Fig. 1 for description of glycan symbols.

In conclusion, we demonstrated that *N-*glycan attachment to proteins is crucial for growth and survival of the red flour beetle, *Tribolium castaneum.* Additionally, we observed for the first time that the global *N-*glycosylation profile changes towards more modified paucimannose glycans during the transition from larval to adult stages. This increased *N-*glycan processing is required for successful pupation, adult eclosion and appendage development (Fig. 7.). Altogether our data provide a new link between protein *N-*glycosylation and execution of metamorphosis, and indicate that genes involved in this process may be promising targets for RNAi-mediated pest control.

## Methods

### Phylogenetic Analyses

*Tribolium* orthologs (from Tcas 4.0 genome assembly) putatively involved in *N-*glycosylation were identified using *Tribolium* BLAST tool (http://bioinf.uni-greifswald.de/blast/tcas/blast.php) with *Drosophila melanogaster* sequences used as queries. Gene functions and specificities were inferred through phylogenetic relationships with known fruit fly and human sequences using MEGA 5.2 and Maximum Likelihood method based on the Poisson correction model. The bootstrap consensus trees were inferred from 1000 replicates^69^.

### Beetle Cultures

For most experiments the unsexed red flour beetles, *T. castaneum* GA-1 strain was used, except for the assessment of RNAi effects on wing discs, in which case the enhancer trap line *pu11* was used. The *pu11* line expresses enhanced yellow fluorescent protein in the eyes, the neurons as well as in the hindwing and elytron discs enabling to track their development. All beetles were cultured at 30 °C and 60% humidity in darkness. Whole wheat flour containing 5% brewer’s yeast was used as a diet.

### Gene Expression Analysis

For gene expression approx. 20 mg of insects at 2^nd^, 4^th^ and 6^th^ (last) larval instar, prepupal, early pupal (0–48 h), late pupal (72–120 h), pharate adult and mature adult stages were homogenized in Trizol and stored at −80 °C. RNA was isolated using Qiagen RNeasy mini kit with on column DNase digestion. cDNA was synthesized with SuperScript™ II Reverse Transcriptase (Invitrogen) using 1 μg of total RNA. qRT-PCR was performed using GoTaq^®^ qPCR Master Mix (Promega) and Bio-Rad CFX Connect robot, cycles included 15 s denaturation at 95 °C, 30 s annealing/extension at 60 °C steps with melt curve analysis at the end of the run. *TcRpS6* and *TcRpL32* were used as reference genes. The final concentration of each primer was 300 nM. Detailed information on primer sequences and the amplification efficiency can be found in Supplementary Table S3. The expression at early larval stage (2^nd^ instar) was used as a baseline expression for further comparisons. The whole experiment was done in three independent biological replications. Quantification of relative transcript abundance using ΔΔCq method, as well as statistical analyses (ANOVA) were performed in qbase+ software (Biogazelle, Zwijnaarde, Belgium). Build-in correction for multiple testing was applied in qbase+. Independent sample t-tests were done using SPSS 22. Primers for qPCR were designed using Primer blast tool (http://www.ncbi.nlm.nih.gov/tools/primer-blast/index.cgi)^70^.

### *N-*glycan Analysis

Approx. 100 mg (wet weight) of 4th instar larvae or 1–2 week old adults were starved for 24 h to exclude the impact of dietary glycoproteins and frozen at −80 °C until use. Whole insects were ground in liquid nitrogen to a fine powder and extracted using 4% SDS, 0.1 M DTT, 0.1 M Tris-Cl pH 7.6. Tryptic peptides were prepared as in ref. 14. Protein extracts were purified by four rounds of washes with 8 M urea in 0.1 M Tris/HCl pH 8.6 (UT) on Amicon Ultra-0.5 mL centrifugal filters (30 kDa cut off). Subsequently, thiol groups were carboxymethylated by incubation of samples with 0.05 M iodoacetamide in UT for 20 mins in the dark. Next proteins were washed twice with UT and twice with 40 mM ammonium bicarbonate. Then porcine trypsin (Thermo Scientific) was added at 1:100 w/w ratio to the protein extracts on filters (5 μg trypsin per 500 μg peptides per filter) and digestion was performed on filters at 37 °C for 16 h. Tryptic peptides were collected by centrifugation and cleaned up using Sep-Pak C18 cartridges (Waters) and 1-propanol/5% acetic acid system. *N-*glycans were released from approx. 2 mg of tryptic peptides using either 0.4 mU PNGase A from almonds (ProGlycAn, Vienna, Austria) in 0.1 M Phosphate-citrate buffer pH 5 or 5 U of PNGase F from *Elizabethkingia meningoseptica* (Sigma-Aldrich) in 40 mM ammonium bicarbonate pH 8 for 16 h. Released glycans were separated from peptides on Sep-Pak C18 cartridges (Waters) using 1-propanol/5% acetic acid system. Samples were desalted using Glycoclean H-cartridge (Prozyme) according to the manufacturer protocol and then evaporated to dryness. Glycans were resuspended in labelling solution (750 mM NaBH_3_CN, 175 mM 2-aminobenzidine in DMSO/acetic acid at 10:3 ratio) and incubated at 65 °C for 2 h. After further purification on Glycoclean S-cartridge (Prozyme) according to the manufacturer protocol, glycans were resuspended in 50% acetonitrile in water for mass spectrometry analysis. Samples were prepared in four biological replicates. MALDI-TOF analyses of larva and adult samples were performed on an UltraFlex II (Bruker) mass spectrometer (4000 laser shots per spectrum) while analyses of RNAi samples were performed on an UltraFlextreme (Bruker) mass spectrometer (500 laser shots per spectrum). The matrix was 2,5-DHB prepared at 20 mg/mL in 50% acetonitrile, 0.1% formic acid. Three mass spectra were recorded for each biological replicate. Laser intensity was optimized for every sample depending on the signal obtained. For each spectrum, the intensities of glycan peaks were normalized on the total glycan signal allowing relative proportions to be determined. To improve sensitivity towards possibly present sialylated oligosaccharides additional larval and adult *N-*glycan preparations were permethylated according to ref. 71. Detected peaks were annotated using GlycoWorkbench 2^72^ based on *N-*glycans described in *Drosophila*. Ambiguous peaks were annotated based on MS/MS fragmentation spectra. Differences between the relative glycan contents were analyzed using one sided t-test in SPSS 22.

### RNA Interference

Gene-specific dsRNAs (without 19–23 bp ‘off-targets’) and primers were designed using the E-RNAi tool http://www.dkfz.de/signaling/e-rnai3//73. Templates were prepared by qPCR using cDNA from 4^th^ instar larvae and specific primers containing T7 promotor sequences at 5′ ends according to standard methods^74^,^75^. dsRNA was produced using MEGAscript^®^ RNAi Kit (Life Technologies). Some dsRNAs were obtained from Eupheria Biotech (Dresden, Germany). At least two genes were silenced for each step of the *N-*glycosylation pathway to further exclude possibility of off-target RNAi effects. A detailed list of primers and dsRNAs can be found in Supplementary Table S3. Fourth-instar larvae weighing 1.2 ± 0.2 mg (randomly assigned to control and treatment groups) anesthetized for 4 min using diethyl ether and injected with 150–250 nL of dsRNA at 1 μg/mL by capillary needle attached to a FemtoJet^®^ injector (Eppendorf). In the case of double RNAi experiments larvae were injected with equal mixtures of two or three dsRNA solutions at total dsRNA concentration of 1 μg/mL. Based on preliminary injections a minimal sample size required to detect 20% growth reduction at 80% power was estimated to consist of 21 larvae (SPSS SamplePower 3). Approx. 60 larvae were individually injected for each gene to ensure sufficient number of larvae in case of high mortality. For each gene or set of injections on a given day an equal number of larvae were injected with dsGFP to serve as a control group, except for the experiment with the *pu11* line in which dsRNA for *TcST6Gal* was used. Silencing of this gene caused no detectable effects. Knockdown efficiency was assessed six days after injection using qRT-PCR as described for gene expression analysis except that three randomly selected individual larvae were used for RNA isolation.

### RNAi Effects

Following the dsRNA injection larvae were allowed to recover for several hours, weighed on a microbalance and placed individually in test tubes containing whole wheat flour diet. Prior to weighing any dead larvae or those with visible cuticle tanning were considered as mechanically damaged due to injection (usually < 10%) and discarded. After 6 days of incubation living larvae were weighted again. Relative larval growth was calculated as a ratio of net larval weight increase to larval weight at day 0. Larvae were then returned to an incubator and examined every other day for mortality, phenotype abnormalities as well as pupation and adult eclosion timing. Images were taken using a Leica SD6 stereomicroscope. Extended depth of field pictures were prepared in FiJi^76^. Briefly, 4–6 pictures taken at different focus levels were aligned using ‘Linear stack alignment with SIFT’ plugin^77^ and finally combined with ‘Extended depth of field’ plugin^78^. The length of pupal elytra was measured as a distance from the joint between T1 tibia and tarsus to the tip of elytra, to avoid errors caused by elytra curvature. The gap between elytra was measured at 0.5 mm from the elytra tip (see the measurement scheme in Supplementary Fig. S6C). To image adult wings, elytra and legs beetles were fixed overnight in 95% ethanol and dissected. For walking speed measurements three- to seven-day-old adults were selected to avoid differences caused by age. Preliminary adult walking speed measurement indicated n = 12 as a minimal number of beetles to detect 20% reduction (SPSS SamplePower 3). If more than 12 adults at appropriate age were available all of them were assayed. Individual beetles were placed in the middle of a square cardboard arena using brush and allowed to adapt for 30 s. Then beetles were placed back in the middle of arena and filmed for at least 30 s using a digital camera. The recorded videos were converted to image sequences at one second intervals and measured using FiJi software. Briefly, images were combined into stack, converted to 8-bit format and threshold was adjusted to isolate pixels representing beetle. Beetle position coordinates were recorded using ‘Analyze Particles’ function (with ‘centroid’ option selected). The average distance between coordinates on adjacent images was calculated as beetle walking distance per second. No blinding was done for the analysis of RNAi effects. Data were analyzed using ANOVA, independent sample t-tests (one-sided) following normality (Shapiro–Wilk) and equality of variance tests (Levene) in SPSS 22.

### Confocal Microscopy

Confocal images of *pu11* prepupae were acquired with a confocal microscope A1R (Nikon), a 405 nm laser and a 450/50 emission filter for cuticle autofluorescence (laser power: 4.0, PMT: 100) and a 488 nm laser and 525/50 nm emission filter for EYFP (laser power: 6.0, PMT: 100), acquisition settings: 6.21 μm pixel size, 1/8 scan speed, 47.3 μm pinhole and Plan Fluor 4x (NA = 0.13) objective were used. All pictures were adjusted identically for brightness and contrast in FiJi software. Ten z stacks were obtained at 50 μm steps and combined to obtain maximum projection images.

## Additional Information

**How to cite this article**: Walski, T. *et al.* Protein *N*-glycosylation and *N*-glycan trimming are required for postembryonic development of the pest beetle *Tribolium castaneum. Sci. Rep.* **6**, 35151; doi: 10.1038/srep35151 (2016).

## Supplementary Material

Supplementary Dataset 1

Supplementary Dataset 2

Supplementary Dataset 3

Supplementary Information

## Figures and Tables

**Figure 2:**
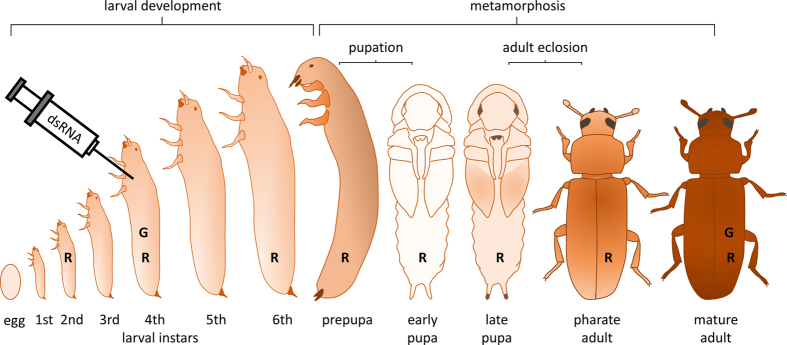
Development of the red flour beetle, *T. castaneum* and timing of the experiments. *T. castaneum* (usually) has 6 larval instars. Sixth-instar larvae develop into immobile, crescent-shaped prepupae and metamorphosis initiates. Prepupae develop into pupae with apparent wing covers and legs. The next stage, pharate adults, eclose from the pupal case and mature in the course of a few days. Syringe indicates injection of dsRNA into 4^th^ instar larvae to analyze developmental consequences of gene knockdowns. ‘**R**’ letters indicate life stages used to isolate RNA for gene expression studies. ‘**G**’ denotes stages used for N-glycan analyses.

**Table 1:** Summary of phenotypes induced by larval RNAi.

Gene silenced	Mortality%				
	larva	pupa	adult	Phenotype description	
*TcDad1*	96.4	1.8	0	Larval growth blocked	Mid-larval mortality
*TcStt3A*	6.0	0	0	Larval growth reduced	
*TcStt3B*	6.9	1.7	8.6	Adult speed reduced	
*TcStt3A + TcStt3B*	95.6	4.4	0	Larval growth blocked	Mid-larval mortality
*TcGCS1*	89.7*	5.2	1.7	Larval growth reduced	Pupation blocked
*TcGCS2*α	90.9*	3.6	0	Larval growth reduced	Pupation blocked
*Tc*α*-Man-Ia*	8.3	10.0	0	Pupal & adult elytra malformed	Adult speed reduced
*Tc*α*-Man-Ib*	5.8	0	1.9	Pupal elytra apart	Adult speed reduced
*Tc*α*-Man-Ia + Tc*α*-Man-Ib*	16.1	73.2	0	Legs, wings and elytra malformed	Adult eclosion blocked
*TcMgat1*	3.7	0	0	Pupal elytra apart & shorter	Adult speed reduced
*Tc*α*-Man-IIa*	1.8	0	0	Pupal elytra apart & shorter	Adult speed reduced
*Tc*α*-Man-IIb*	5.5	0	0	Pupal elytra apart & shorter	
*Tc*α*-Man-IIa + Tc*α*-Man-IIb*	1.9	0	0	Pupal elytra apart & shorter	Adult speed reduced
*Tcfdl*	1.5	0.0	4.5	Pupal elytra apart & shorter	Adult speed reduced
*TcMgat2*	3.1	0	0	none	
*TcMgat4*	7.1	0	0	none	
*TcMgat1 + TcMgat4*	4.0	0	0	Pupal elytra apart	Adult speed reduced
*TcMgat1 + TcMgat2 + TcMgat4*	0	0	0	Pupal elytra apart & shorter	Adult speed reduced
*TcFucT6*	0	0	0	none	
*TcFucTA/C*	0	0	0	none	
*TcFucTA*	5.3	0	0	none	
*Tcβ4GalNAcTA*	0	0	0	Larval growth reduced	
*Tcβ4GalNAcTB*	4.5	0	4.5	none	
*Tcβ4GalNAcTA + Tcβ4GalNAcTB*	4.2	0	0	Larval growth reduced	
*TcST6Gal*	0	3.3	0	none	
*TcCsas*	0	0	0	none	

^*^Majority of beetles died during prepupa to pupa transition.
