# Supplementary material for: Protein N-glycosylation and N-glycan trimming are required for postembryonic development of the pest beetle Tribolium castaneum
Source: Sci Rep. 2016 Oct 12;6:35151. doi: 10.1038/srep35151 (PMC5059678; doi:10.1038/srep35151)
Supplement: Supplementary Information [file srep35151-s4.pdf]

Protein *N*-glycosylation and *N*-glycan trimming are required for postembryonic development of the pest beetle *Tribolium castaneum*

Tomasz Walski[1,2], Els J. M. Van Damme[2], Nicolas Smargiasso[3], Olivier Christiaens[1], Edwin De Pauw[3], Guy Smagghe[1]

[1]Laboratory of Agrozoology, Department of Crop Protection, Faculty of Bioscience Engineering, Ghent University, Ghent, Belgium

[2]Laboratory of Biochemistry and Glycobiology, Department of Molecular Biotechnology, Faculty of Bioscience Engineering, Ghent University, Ghent, Belgium

[3]Mass Spectrometry Laboratory, GIGA-Research, University of Liege, Liege, Belgium

Supplementary information

Supplementary Table 1. Developmental expression changes of genes putatively involved in N-glycan attachment and processing in *T. castaneum*

		Stage with the highest expression vs 4th instar larvae			Mature adults vs 4th instar larvae	
**Gene**	**ANOVA P**	**Stage**	**Expression ratio (95% CI)**	**t-test P**	**Expression ratio (95% CI)**	**t-test P**
TcDad1	<0.001	Early pupae	1.87 (1.34-2.56)	<0.001	1.08 (0.78-1.49)	0.262
TcStt3A	0.011	Early pupae	2.35 (1.11-4.35	0.029	1.15 (0.58-2.26)	0.123
TcStt3B	0.547	Late pupae	1.32 (0.53-3.13)	0.998	1.01 (0.42-2.45)	0.472
TcGCS1	0.001	Late pupae, pharate adults	2.86 (1.34-6.45)	0.018	2.25 (1.07-5.15)	0.032
TcGCS2α	0.013	Early pupae	3.03 (1.21-11.76)	0.018	2.30 (0.91-8.85)	0.029
Tcα-Man-Ia	<0.001	Late pupae	1.74 (1.21-2.49	0.002	1.46 (1.01-2.07)	0.031
Tcα-Man-Ib	<0.001	Pharate adults	2.97 (1.66-5.85)	0.001	2.53 (1.39-4.90)	0.012
TcMgat1	0.025	Late pupae	1.86 (0.92-4.24)	0.111	1.35 (0.66-3.06)	0.128
Tcα-Man-IIa	<0.001	Early pupae	3.88 (1.80-8.26)	0.004	2.33 (5.18-1.12)	0.008
Tcα-Man-IIb	<0.001	Early pupae	3.59 (1.71-7.94)	0.002	2.23 (1.05-4.90)	0.022
Tcfdl	0.009	Early pupae	4.86 (1.21-23.26)	<0.001	1.37 (0.26-5.05)	0.316
TcFucT6	0.003	Early pupae	4.14 (1.63-16.39)	<0.001	2.55 (1.03-10.31)	0.011
TcFucTA	0.001	4th inst. larvae	1.00	-	0.74 (0.23-2.37)	0.101
TcFucTA/C	<0.001	Late pupae	8.11 (3.75-15.63)	<0.001	2.55 (1.24-5.21)	0.006
TcFucTB	<0.001	Early pupae	4.56 (2.59-8.26)	<0.001	2.35 (1.31-4.20)	0.012
TcFucTC	<0.001	Prepupae	2.68 (1.64-4.59)	<0.001	1.63 (0.97-2.71)	0.059
TcMgat2	<0.001	Early pupae	4.77 (1.87-16.13)	0.004	2.65 (1.07-9.26)	0.007
TcMgat4	0.004	Late pupae	9.21 (2.18-142.86)	0.003	4.08 (1.01-62.50)	0.006
TcβGalNAcTA	<0.000	Early pupae	10.24 (2.74-38.46)	0.003	2.57 (0.75-10.31)	0.037
TcβGalNAcTB	<0.000	Early pupae	2.15 (1.41-3.28)	0.001	1.60 (1.05-2.44)	0.016
TcST6Gal	<0.000	Late pupae	3.55 (2.18-6.06)	<0.001	2.77 (1.71-4.76)	0.001
TcCsas	<0.000	Early pupae	6.60 (2.96-16.13)	0.001	5.65 (2.61-14.29)	0.002

ANOVA P – p-value obtained using ANOVA in qbase+ software to reveal life stage dependent changes in expression. T-test P – p-value obtained via independent sample t-test to compare expression between 4th instar larvae and life stage with highest expression or between 4th instar larvae and mature adults. All values are averages of 3 biological replications. 95% CI are 95% confidence intervals for expression ratio between indicated life stages.

Supplementary Table 2. N-glycan content

	Measured m/z $[M+Na^{+}]$	Glycan content [% total profile] Larva	Adult	L vs. A P	α-Man-Ia RNAi Adult	A vs. A^{RNAi} P	Deduced structure	Composition
1.	**891.3**	3.5±0.7	6.1±0.9	0.004	3.8±0.1	0.007	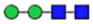	HexNAc2Hex2
2.	**1021.4**	0.2±0.4	1.2±0.2	0.005	0.1±0.1	0.001	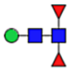	HexNAc2HexdHex2
3.	**1037.4**	11.5±3.1	21.9±5.8	0.013	4.6±0.3	0.003	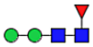	HexNAc2Hex2dHex
4.	**1053.3**	3.4±0.7	4.7±0.4	0.015	4.8±0.2	0.294	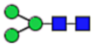	HexNAc2Hex3
5.	**1183.5**	7.3±1.3	11±3.8	0.063	1.8±0.2	0.007	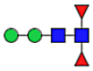	HexNAc2Hex2dHex2
6.	**1199.4**	4.0±0.8	8.4±1.1	0.001	1.8±0.2	0.000	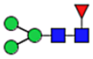	HexNAc2Hex3dHex
7.	**1215.5**	2.4±0.5	3.5±0.2	0.010	4.7±0.2	0.001	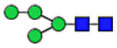	HexNAc2Hex4
8.	**1256.5**	0.8±0.3	0.7±0.4	0.297	0.1±0	0.034	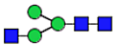	HexNAc3Hex3
9.	**1345.5**	1.2±0.4	1.2±0.2	0.391	0.2±0	0.000	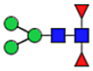	HexNAc2Hex3dHex2
10.	**1361.5**	0.2±0.2	0.7±0.3	0.029	0.1±0	0.023	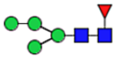	HexNAc2Hex4dHex
11.	**1377.5**	3.3±0.4	4.5±0.3	0.003	5.5±0.3	0.013	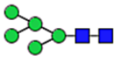	HexNAc2Hex5
12.	**1402.5**	1.0±0.3	1.4±0.3	0.105	0.4±0	0.003	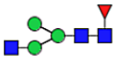	HexNAc3Hex3dHex
13.	**1418.4**	0.3±0.1	0.1±0.1	0.016	<0.1	0.082	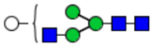	HexNAc3Hex4
14.	**1539.5**	2.9±0.3	2.6±0.4	0.231	5.6±0.3	0.000	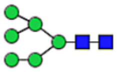	HexNAc2Hex6
15.	**1548.5**	0.2±0.2	0.4±0.3	0.139	<0.1	0.049	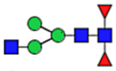	HexNAc3Hex3dHex2
16.	**1580.5**	0.3±0.1	0.2±0.1	0.161	n.d.	-	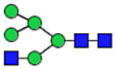	HexNAc3Hex5
17.	**1605.5**	0.2±0.1	0.3±0.2	0.160	<0.1	0.039	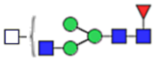	HexNAc4Hex3dHex
18.	**1701.6**	3.9±0.4	2.6±0.5	0.005	11.0±0.4	<0.001	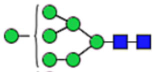	HexNAc2Hex7
19.	**1863.6**	6.9±0.7	3.8±0.9	0.002	15.9±0.6	<0.001	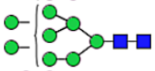	HexNAc2Hex8
20.	**2025.7**	40.7±7.4	22.0±6.0	0.008	35.5±1.5	0.010	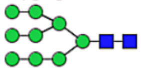	HexNAc2Hex9
21.	**2187.7**	5.8±1.4	2.2±0.4	0.004	4.0±0.5	0.005	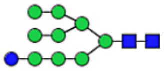	HexNAc2Hex10

[M+Na]+ indicate measured masses of sodiated 2-AB-glycan ions. Glycan content values are means of glycan abundance relative to all glycans detected ± standard deviation (n=4). P's were calculated using independent sample t-test (SPSS 22).

Supplementary Table S3. Primers used for qRT-PCR analysis of gene expression.

gene	*Tribolium* accession number		qRT-PCR primer sequence	Amplicon length	Efficiency (%)
TcDad1	TC016366	F<br>R	CCACAGGTGACTTCAGTTATCG<br>AAAAGTGCCCACAAGACAGC	147	92.5
TcStt3A	TC010433	F<br>R	TGTGCTACTACCGCTTTGGG<br>ATACGCACGAGCCAGTGTTC	148	94.7
TcStt3B	TC009183	F<br>R	CCCGCTTCATGTGTTTGTCC<br>TCGCTGGTACGAATGGGTTG	141	101.1
TcGCS1	TC011354	F<br>R	ATCGGCCCTCAGTAACATGC<br>AAACTTCGAGACGGGACTGC	132	95.5
TcGCS2α	TC032148	F<br>R	GGGAAGGGGAACAAGAGGTG<br>TCAACACCCACTGCTTCAGG	103	97.6
Tcα-Man-Ia	TC011089	F<br>R	AATGCAAGCTGTCCTCCAAC<br>CAGACCACCCGAAAAACAAC	124	97.6
Tcα-Man-Ib	TC014283	F<br>R	ACGTGCTATTTGCCTGGAAC<br>TTCGGGTGCTAGAAATGTGG	83	98.9
TcMgat1	TC009001	F<br>R	ACCAGGAGTTGAAAGAGGGC<br>TACCCTTGTACGCAGTTCGG	147	94.1
Tcα-Man-IIa	TC009186	F<br>R	CAAAGCGAAGAAAGGAGTGG<br>ACGATCGGTCCTTGAATCAC	104	97.2
Tcα-Man-IIb	TC002991	F<br>R	GGAGTTCCCCTGTGATACGC<br>ACTCACAGCACAGTCGTAGC	124	95.2
Tcfdl	TC009779	F<br>R	CCCAGAGTTTTCCTTTGGTG<br>CTCTGATTCGGGCATATTCG	123	91.6
TcFucT6	TC008521	F<br>R	ATTCGTTGCGTGGAGACCAG<br>TGGGCTCTTCTTTCGCACTC	117	103.5
TcFucTA/C	TC005825	F<br>R	ACGTCACGGAGAAGCTTTGG<br>CCTGTAACTCGCCACGGAAG	83	94.2
TcFucTA	TC014343	F<br>R	CACCCTCCAGTTTCCAGACC<br>CGACCAGATCGCTGTCTCTG	136	92.0
TcFucTB	TC007385	F<br>R	ACAACGAACAACTGCTCCAC<br>GATGTAGTCGTCGCAAATGG	80	91.6
TcFucTC§	TC008651/2	F<br>R	CGACAAACCAACCTTTCTGG<br>GGCTTTGGAACATTGGTGTC	71	90.6
TcMgat2	TC001867	F<br>R	CCCATACTCGATCCAAACACATCC<br>GAGGGCATTGTTGCATTTCTGG	112	97.7
TcMgat4	TC003870	F<br>R	GGTGCACTACAAACCTTCGC<br>ATTCCTGAGACGACTTCCGC	147	94.3
Tcβ4GalNAcTA	TC006388	F<br>R	GCAAGATACACGATGCTGACG<br>TCCTTGTCGAAGCGTTTGGG	92	91.0
Tcβ4GalNAcTB	TC006987	F<br>R	CGGTTGTGTGAGCAAACCAAG<br>CGATGAAATCGCCACAACTCC	103	97.1
TcST6Gal	TC014265	F<br>R	CTCACCGAGCAGACTTAGGAG<br>GCGTTTAGTGACCCTGGTTG	124	94.0
TcCsas	TC015995	F<br>R	CAAGGGAAAGGAGTGTGTCTTC<br>CTTGTCTTCGTGGTCGATTG	119	92.5
TcRpS6	TC010830	F<br>R	GAATATCGGGCGGGAACGAC<br>TGCACCCTCTAACTGACTTGC	150	95.7
TcRpL32	TC006106	F<br>R	ACCAGTCTGACCGTTATGGC<br>CGTAGCCAATGTTGGGCATC	114	94.2

§ - primer for FucTC was designed to detect both FucTC paralogs TC008651 and TC008652. The paralogs share 87% identical aminoacids.

Supplementary Table S4. Primers used for dsRNA synthesis and dsRNAs obtained from Eupheria

Target gene	*Tribolium* accession number		Primer sequence	dsRNA length
TcDad1	TC016366	F	taatacgactcactatagggTGCCACAGGTGACTTCAGTT	367
		R	taatacgactcactatagggATGTAGGACCACATGGGCG	
TcGCS1	TC011354	F	taatacgactcactatagggCAACAACCAATCGTGAGCTG	374
		R	taatacgactcactatagggGACCAATCGGTATTCGGGAT	
Tcα-Man-Ia	TC011089	F	taatacgactcactatagggCTTGGGGCAAGAACGAACT	381
		R	taatacgactcactatagggCCAGTCTGTGTCTGAAACGC	
Tcα-Man-Ib	TC014283	F	taatacgactcactatagggCTAGTCTCGACCGAATCCGA	385
		R	taatacgactcactatagggTGTCAATCGAGTCAACAATGC	
Tcα-Man-IIa	TC009186	F	taatacgactcactatagggTGTCCATGGAAAGTGCCC	365
		R	taatacgactcactatagggAAAAATCGCCCGATAACGA	
Tcα-Man-IIb	TC002991	F	taatacgactcactatagggGGCCATAGGCCTCACAATAA	423
		R	taatacgactcactatagggTTCATTATCTGGCGGGAGAC	
TcFucTA/C	TC005825	F	taatacgactcactatagggAATCGAAATTTTCAGCGTCG	435
		R	taatacgactcactatagggCACAATTCGAGCCCATGAT	
TcFucTA	TC014343	F	taatacgactcactatagggATGCCTCCGCGACTCTCT	378
		R	taatacgactcactatagggGGCAAGAATCACTTTCTCCG	
Tcβ4GalNAcTA	TC006388	F	taatacgactcactatagggACGCTCCATCTCAAGAAAGC	500
		R	taatacgactcactatagggTCTTCCGGCAACAAATCAAT	
TcST6Gal	TC014265	F	taatacgactcactatagggACGCTCATGATTTGGTTTTG	374
		R	taatacgactcactatagggCAAAAACCCCGACGATAGTG	
TcCsas	TC015995	F	taatacgactcactatagggTCTTTTTCATGCAAATCCAGC	395
		R	taatacgactcactatagggACGTACACTGCACCAAAGCA	
			**Eupheria catalog number**	
TcStt3A	TC010433		iB_01689	479
TcStt3B	TC009183		iB_01492	497
TcGCS2α	TC032148		iB_00458	501
TcMgat1	TC009001		iB_01454	489
Tcfdl	TC009779		iB_04695	490
TcFucT6	TC008521		iB_04509	481
TcMgat2	TC001867		iB_03261	482
TcMgat4	TC003870		iB_00631	483
Tcβ4GalNAcTB	TC006987		iB_06724	391

taatacgactcactataggg – is a T7 promoter sequence. F- forward, R - reverse.

Supplementary Figure 1. Phylogeny of proteins putatively involved in N-glycan attachment and processing

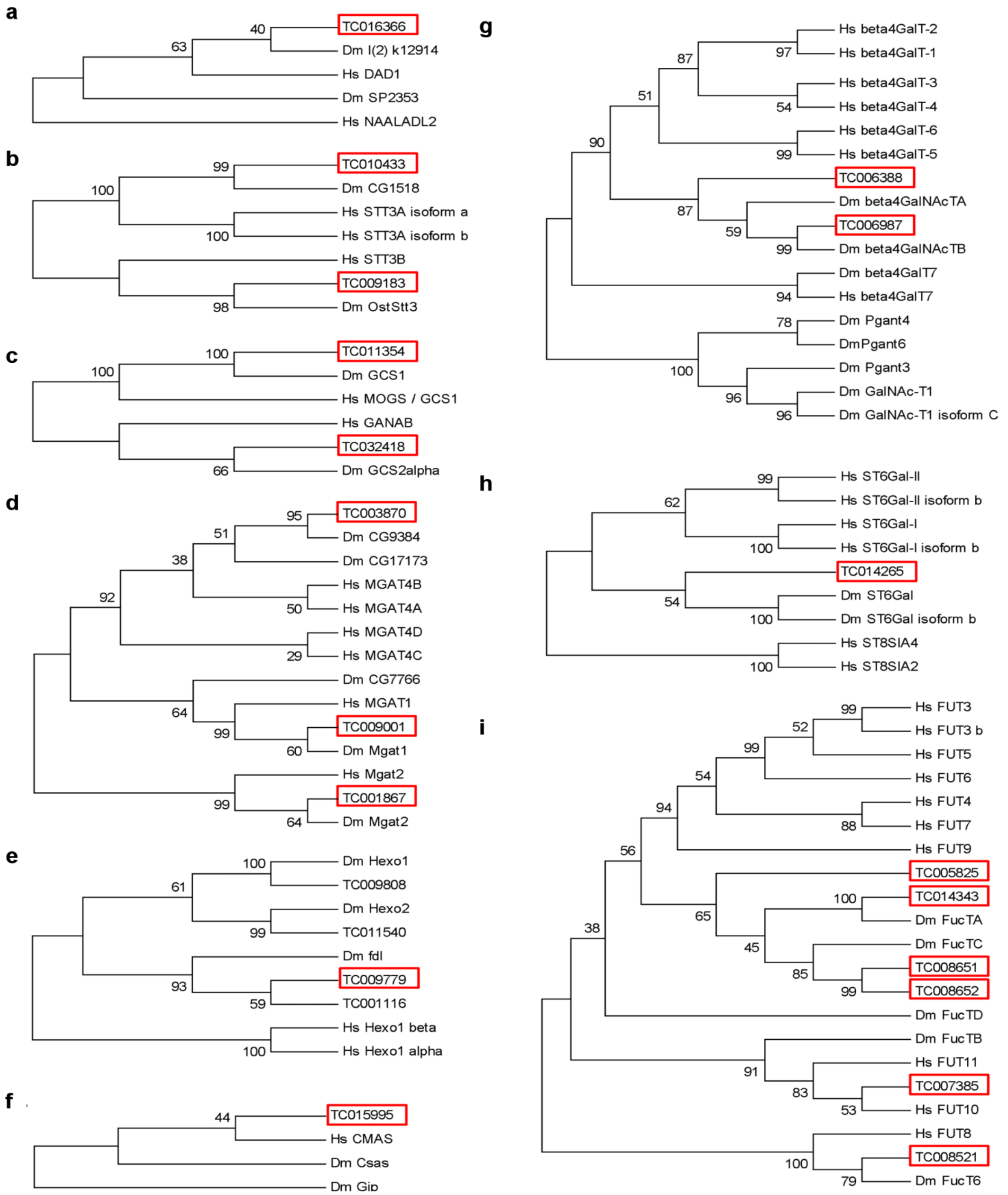

Fig. S1. Phylogeny of proteins putatively involved in N-glycan attachment and processing in *Tribolium castaneum*. The evolutionary history was inferred by using the Maximum Likelihood method based on the Poisson correction model. Branches corresponding to partitions reproduced in less than 50% bootstrap replicates are collapsed. The percentages of replicate trees in which the associated taxa clustered together in the bootstrap test (1000 replicates) are shown next to the branches. Hs - *homo sapiens*, Dm –

Drosophila melanogaster, TC0##### are putative *Tribolium castaneum* orthologs obtained from http://bioinf.uni-greifswald.de/gb2/gbrowse/tcas4. Tribolium orthologs studied in this paper are in brackets. (**a**) DAD1 orthologs. RefSeq accessions: Hs DAD1 - NP 001335.1; Dm lethal (2) k12914 - NP 609222.1; Dm SP2353 - NP 611082.2; Hs NAALDL2 - XP 011510919.1. The latter two sequences are remotely related and are used as outgroups to improve the quality of tree. (**b**) Orthologs of STT3 subunits. RefSeq accessions: Hs STT3A - NP 689926.1, NP 001265433.1; Hs STT3B - NP 849193.1; Dm CG1518 - NP 608425.1; Dm OstStt3 - NP 524494.1. (**c**) Orthologs of α-glucosidases. RefSeq accessions: Hs MOGS/GCS1 - NP_001139630.1; Hs GANAB - NP_001265121.1; Dm GCS1 - NP_001245621.1; Dm GCS2α - NP_652145.1. (**d**) Orthologs of GlcNAc transferases, RefSeq accessions: Hs MGAT1 - NP 002397.2; Hs MGAT2 - NP 002399.1; Hs MGAT4A - NP 036346.1; Hs MGAT4B - NP 055090.1; Hs MGAT4C - NP 037376.2; Hs MGAT4D - NP 001264282.1; Dm Mgat1 - NP 525117.2; Dm Mgat2 - NP 001263096.1; Dm CG9384 - NP 648720.3; Dm CG17173 - NP 648721.1; Dm CG7766 - NP 001138180.1. (**e**) Orthologs of N-acetylglucosaminidases, RefSeq accessions: Hs Hexo1α - NP 000511.1; Hs Hexo1β - NP 000512.1; Dm Hexo1 - NP 728975.1; Dm Hexo2 - NP 525081.1; Dm fdl - NP 001286351.1. (**f**) Orthologs of CMP-sialic acid synthase, RefSeq accessions: Hs CMAS - NP 061156.1; Dm Csas - ACH92492.1; Dm Gip - NP 511106.1 (distantly related sequence used as an outgroup for tree construction). (**g**) Orthologs of Gal/GalNAc transferases RefSeq accessions: Hs beta4GalT-2 isoform b - NP 003771.1; Hs beta4GalT-2 - NP 085076.2; Hs beta4GalT-1 - NP 001488.2; Hs beta4GalT-3 - NP 003770.1; Hs beta4GalT-4 - NP 003769.1; Hs beta4GalT-6 - NP 004766.2; Hs beta4GalT-5 - NP 004767.1; Hs beta4GalT7 - NP 651319.2; Dm beta4GalNAcTA - NP 610946.1; Dm beta4GalNAcTB - NP 651657.1; Dm beta4GalT7 - NP 009186.1; Dm Pgant4 - NP 001137779.1; Dm Pgant6 - NP 647749.2; Dm GalNAc-T1 - NP 611043.1; Dm GalNAc-T1 isoform C - NP 001286461.1. (**h**) Orthologs of sialic acid transferases, RefSeq accessions: Hs ST6Gal-II - NP 115917.1; Hs ST6Gal-II isoform b - NP 001135824.1; Hs ST6Gal-I - NP 003023.1; Hs ST6Gal-I isoform b - NP 775324.1; Hs ST8SIA4 - NP 005659.1; Hs ST8SIA2 - NP 006002.1; DmSt6Gal - NP 726474.1; DmSt6Gal isoform b - NP 523853.1. (**i**) Orthologs of Fucosyltransferases, RefSeq accessions: Hs FUT3 - NP 000140.1; Hs FUT5 - NP 002025.2; Hs FUT6 - NP 000141.1; Hs FUT4 - NP 002024.1; Hs FUT7 - NP 004470.1; Hs FUT9 - NP 006572.2; Hs FUT11 - NP 775811.2; Hs FUT10 - ref|NP 116053.3; Hs FUT8 - NP 835368.1; Dm FucTA - NP 648754.2; Dm FucTC - NP 001036320.3; Dm FucTD - NP 612107.1; Dm FucTB - |NP 609288.4; Dm FucT6 - NP 572740.1.

Supplementary Figure 2. Phylogeny of putative *Tribolium* α-mannosidases

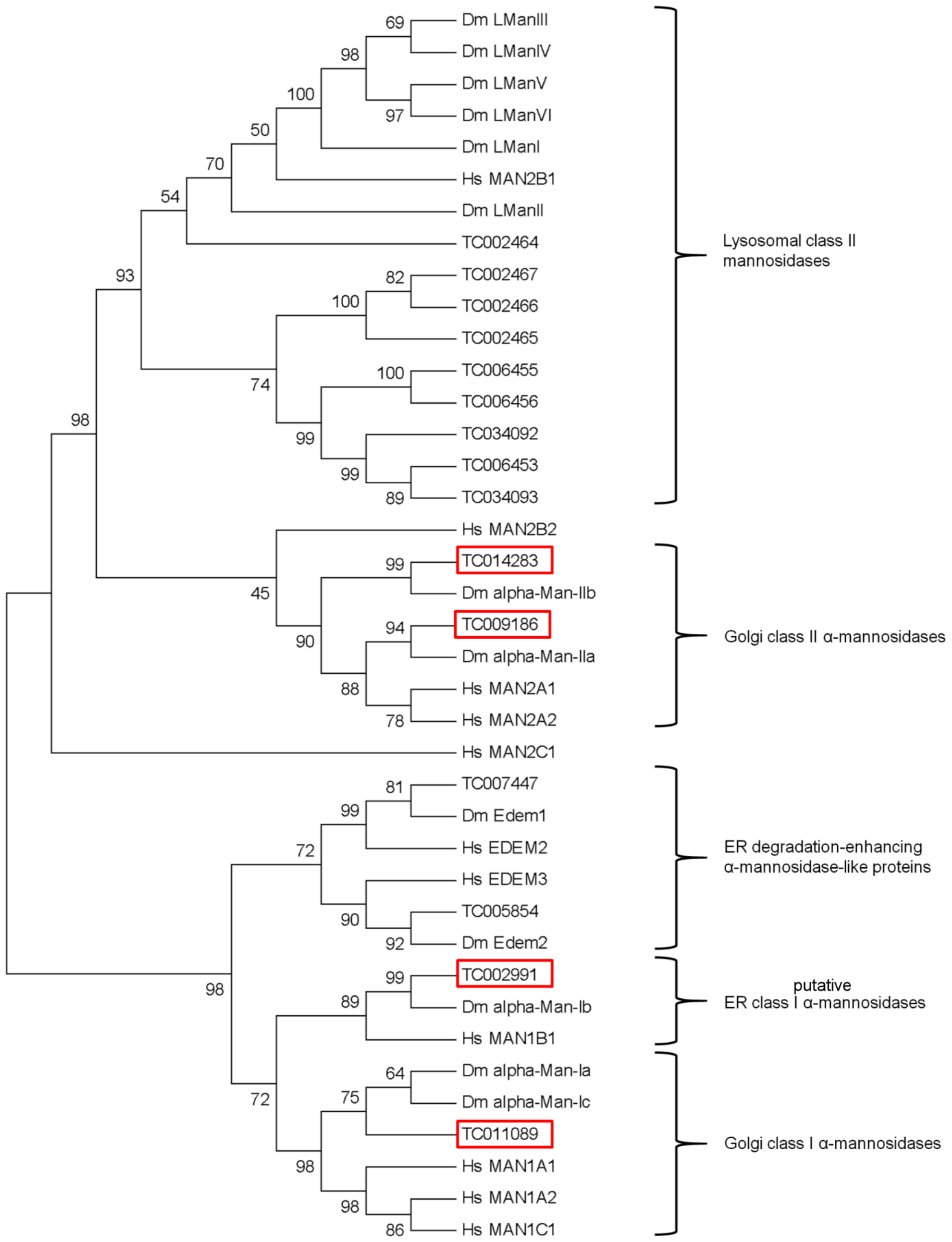

Fig. S2. Phylogeny of putative *Tribolium* α-mannosidases. The evolutionary history was inferred by using the Maximum Likelihood method based on the Poisson correction model. Branches corresponding to partitions reproduced in less than 50% bootstrap replicates are collapsed. The percentage of replicate trees in which the associated taxa clustered together in the bootstrap test (1000 replicates) are shown next to the branches. Hs - *homo sapiens*, Dm – *Drosophila melanogaster*, TC0##### are putative *Tribolium castaneum*

orthologs obtained from http://bioinf.uni-greifswald.de/gb2/gbrowse/tcas4. Tribolium orthologs studied in this paper are in brackets. RefSeq accessions: Hs MAN2B1 - NP 000519.2; Hs MAN2B2 - AAH94773.1; Hs MAN2A1 - NP 002363.2; Hs MAN2A2 - NP 006113.2; Hs MAN2C1 - NP 006706.2; Hs MAN1B1 - NP 057303.2; Hs MAN1A1 - NP 065112.1; Hs MAN1A2 - NP 006690.1; Hs MAN1C1 - NP 005898.2; Hs EDEM2 - NP 060687.2; Hs EDEM3 - XP 005245556.1; Dm LManIII NP 609250.2; Dm LManIV - NP 609251.2; Dm LManV - NP 609252.1; Dm LManVI - NP 609253.1; Dm LManI - NP 609407.1; Dm LManII - NP 609408.1; Dm α-Man-IIb - NP 650494.2; Dm α-Man-IIa - NP 524291.2; Dm Edem1 - NP 001259152.1; Dm Edem2 - NP 609611.1; Dm α-Man-Ia - NP 727408.2; Dm α-Man-Ib - NP 651667.1; Dm α-Man-Ic - NP_733331.1.

Supplementary Figure 3. Examples of MALDI-MS spectra of *Tribolium* N-glycans.

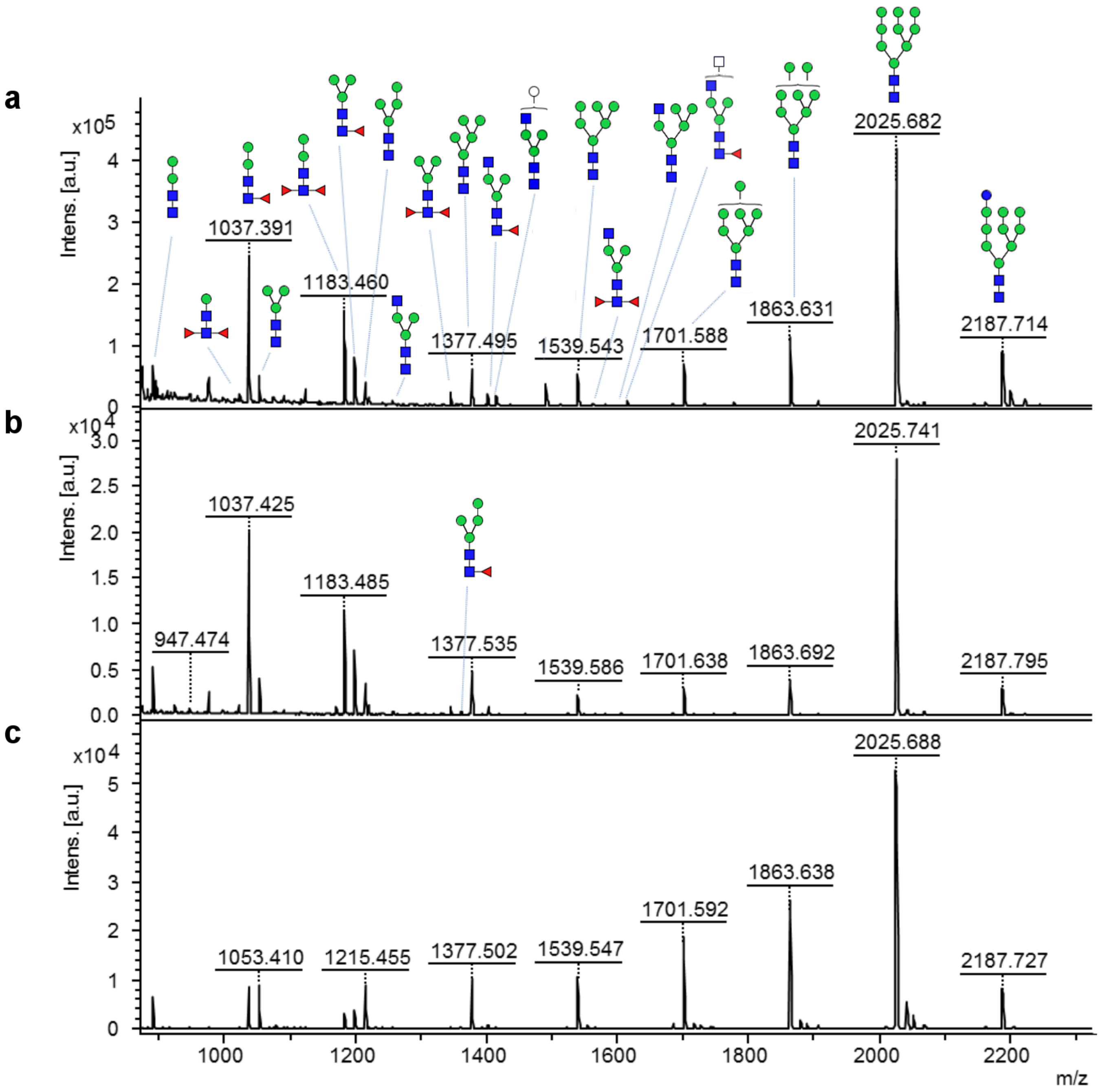

Fig. S3. Examples of MALDI-MS spectra of *Tribolium* N-glycans. Proteins were extracted from whole 4th instar larvae or mature adults. N-glycans were released using PNGaseA, labeled with 2-AB and analyzed by MALDI-MS using DHB matrix. **(a)** larval N-glycans. (**b**) adult N-glycans. (**c**) *Tcα-Man-Ia* RNAi adult N-glycans. Source files for the representative spectra are included in the Supplementary Files online.

Supplementary Figure 4. Efficiency of RNAi mediated gene knockdown.

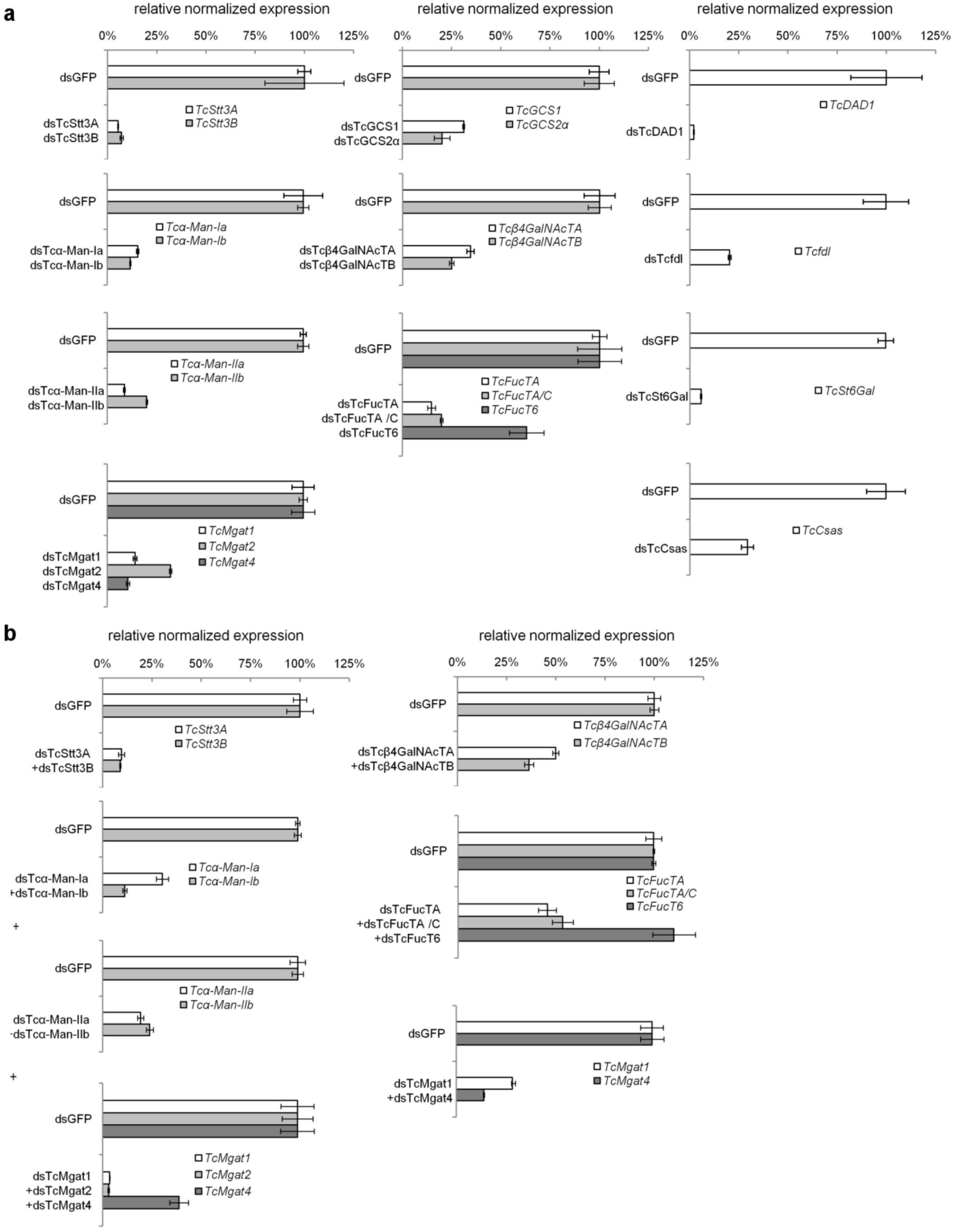

Fig. S4. Efficiency of RNAi mediated gene knockdown. 4th instar larvae were injected with appropriate dsRNAs to induce gene silencing or with dsGFP in case of control. Total RNA was isolated 6 days post injection. Relative gene expression was quantified with qRT-PCR using *TcRpL32* and *TcRpS6* as reference genes and normalized to control. Data are averages ± SD (n=3). (**a**) Single knockdown experiments. (**b**) Multiple knockdown experiments performed by injection of mixture of indicated dsRNA's.

Supplementary Figure 5. RNAi of genes involved in GlcNAc attachment and removal cause minor developmental defects.

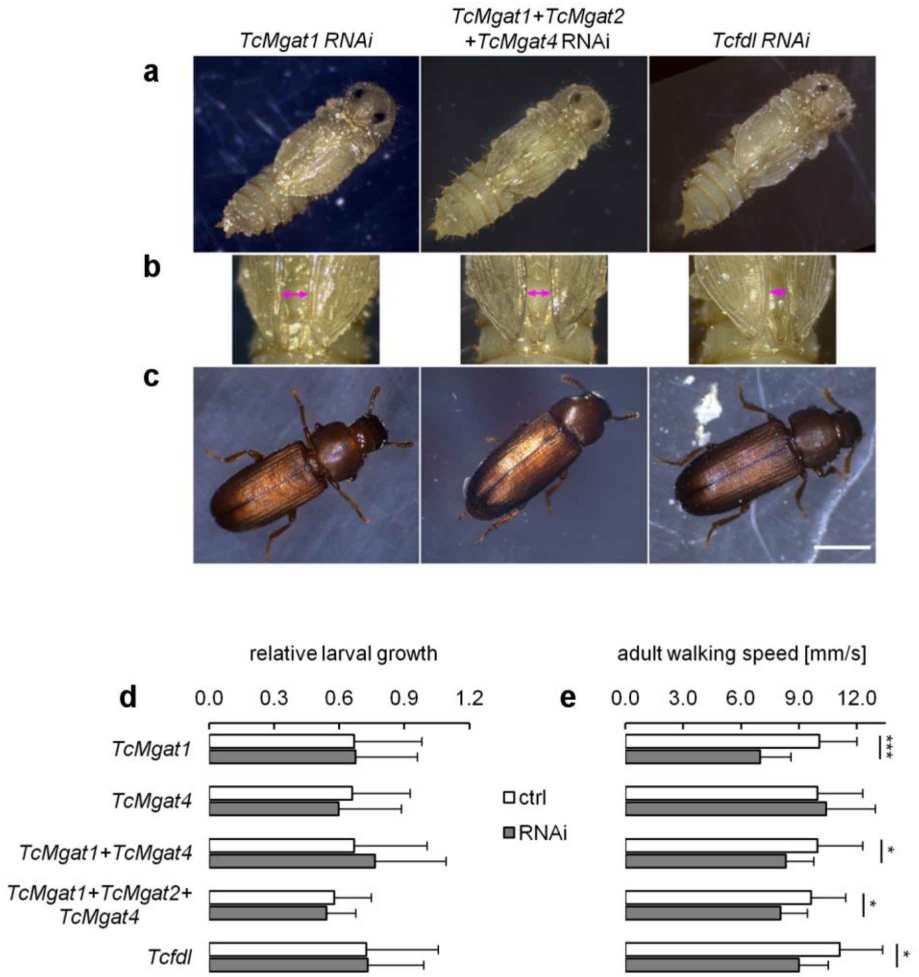

Fig. S5. RNAi of genes involved in GlcNAc attachment and removal cause minor developmental defects. 4th instar larvae were injected with appropriate dsRNAs to induce gene silencing or with dsGFP in case of control. (**a**) and (**b**) RNAi of *TcMgat1* and *Tcfdl* caused minor disruption of pupal elytra but not of (**c**) adult morphology and (**d**) larval growth (n=57, 57, 58, 58, 69). (**e**) Silencing of *TcMgat1* and *Tcfdl* reduced adult mobility (n=20, 12, 12, 12, 12). Double RNAi of *TcMgat1* and *TcMgat4* as well as triple RNAi of *TcMgat1*, *TcMgat2* and *TcMgat4* did not cause any increase in phenotype severity. Scale bar is 1 mm in (**a**) and (**c**) and 0.5 mm in (**b**). Error bars are SD, asterisks indicate statistically significant differences: *** P<0.001, ** P<0.01, * P<0.05 (t-test).

Supplementary Figure 6. RNAi of genes involved in N-glycan processing affect pupal elytra morphology.

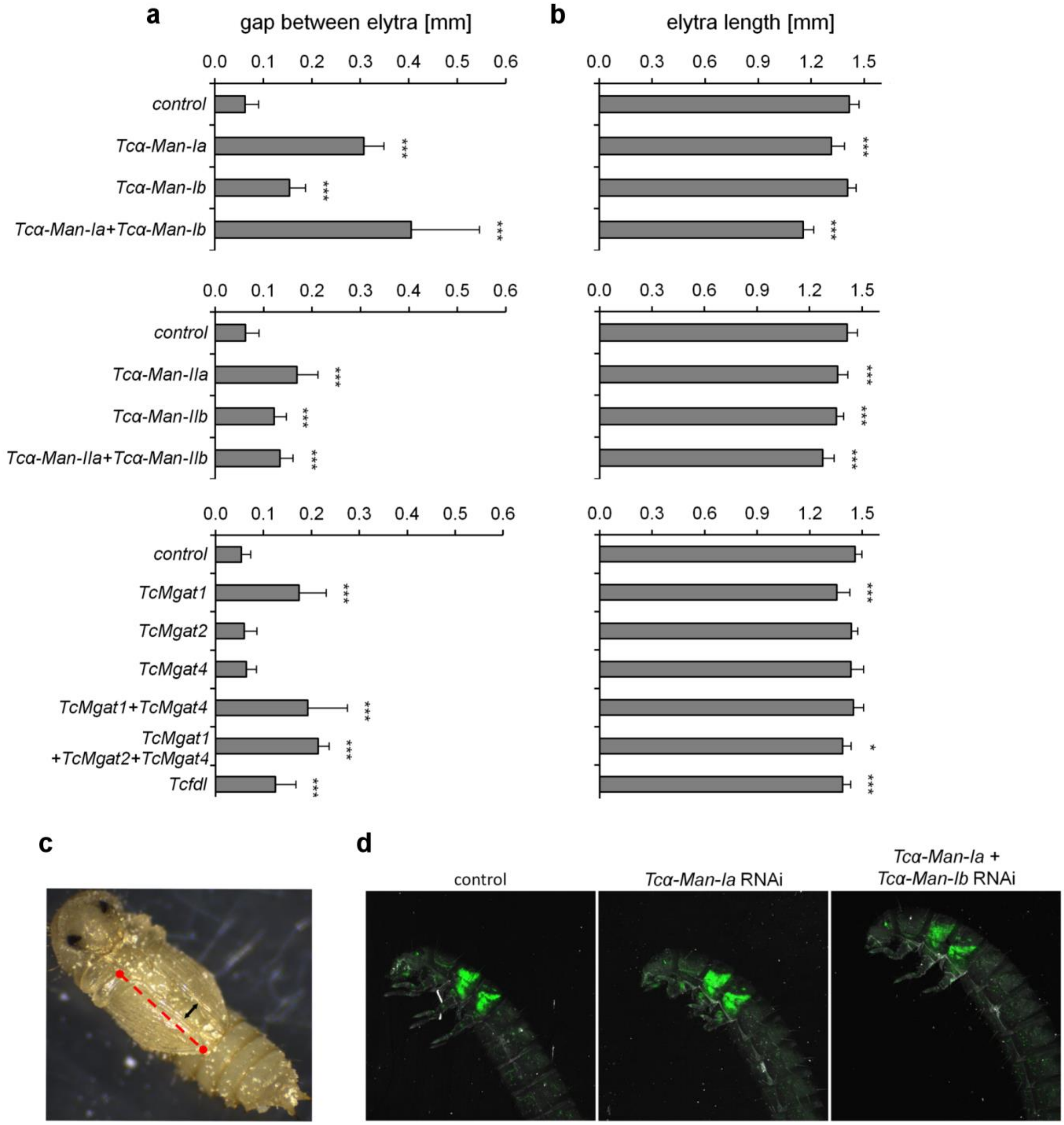

Fig. S6. RNAi of genes involved in N-glycan processing affect pupal elytra morphology. (**a**) Knockdown effects on elytra position. Elytra gap is enlarged in case of RNAi of each of the genes involved in N-glycan processing towards paucimannose glycans (all four α-mannosidases, *TcMgat1* and *Tcfdl*) and not after RNAi of *TcMgat2* and *4* which are involved in complex glycan synthesis. (**b**) Effects of N-glycan processing genes RNAi on pupal wing length (n=11-17). (**c**) Scheme of elytra length (red dashed line) and gap (black double-headed arrow) measurement. To avoid errors caused by elytra curvature, the length was measured as distance from the joint between T1 tibia and tarsus to the tip of elytra. The gap between elytra was measured at 0.5 mm from the elytra tip. Error bars are SD, asterisks indicate statistically significant differences compared to control: *** P<0.001, ** P<0.01, * P<0.05 (t-test). (**d**) RNAi of class I α-mannosidases had no clear effect on development of wing and elytra wing discs in *pu11* strain. This strain expresses EYFP in wing and elytron primordia, eyes and neurons. Maximum projection confocal images are shown: green - EYFP, white – cuticle autofluorescence.

Supplementary Figure 7. RNAi of class I α-mannosidases affect adult appendage development.

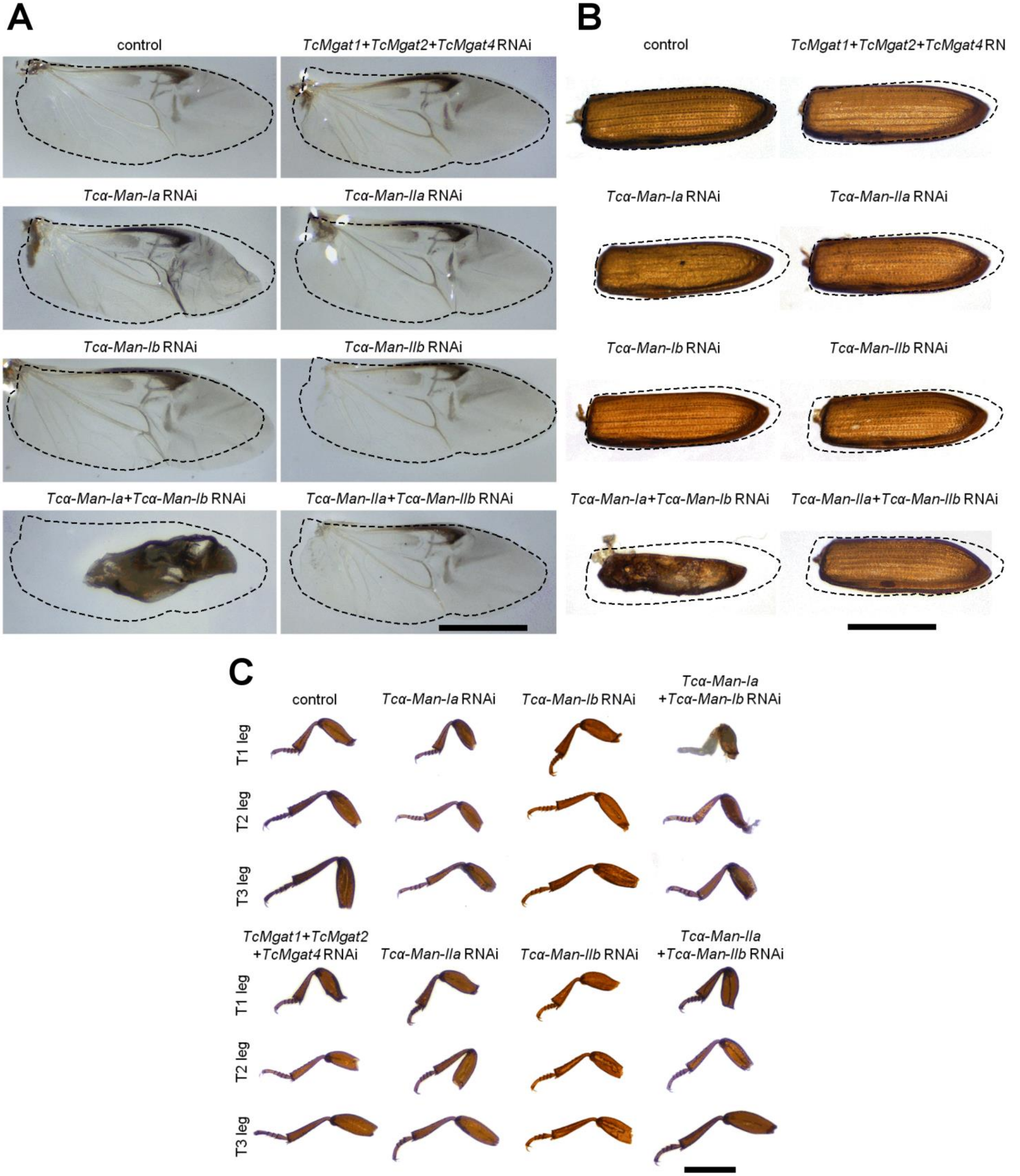

Fig. S7. RNAi of class I α-mannosidases affect adult appendage development. (**a**) RNAi effects on adult wings, (**b**) adult elytra and (**c**) adult legs. Knockdown of *Tcα-Man-Ia* caused minor malformations: wrinkled and smaller wings and elytra. Double knockdown of *Tcα-Man-Ia* and *Tcα-Man-Ib* completely blocked wing and elytra development and interfered with proper leg sclerotization (especially visible on T1 legs). Other knockdowns caused no obvious effects. Dashed line indicate shape of wing and elytron of control beetles. Scale bars in (A) and (B) are 1 mm. Scale bar in (C) is 0.5 mm.

Supplementary Figure 8. Silencing of genes potentially involved in complex glycan synthesis and sialylation caused minor or no effects.

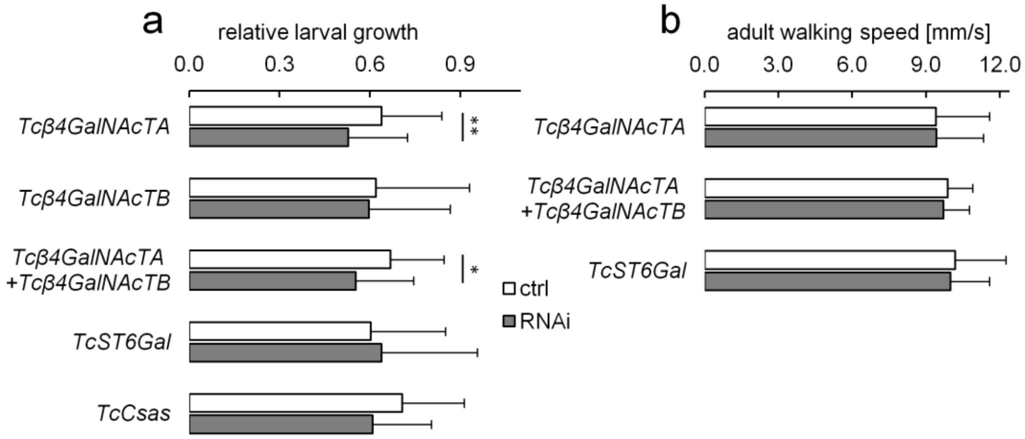

Fig. S8. Silencing of genes potentially involved in complex glycan synthesis and sialylation caused minor or no effects. (**a**) Only RNAi of *Tcβ4GalNAcTA* caused minor reduction of larval growth (n=49-63). (**b**) There was no effect on adult movement speed (n= 15-25). Error bars are SD, asterisks indicate P<0.001 (***), P<0.01 (**), P<0.05 (*).
